# The natural compound GL22, isolated from *Ganoderma* mushrooms, suppresses tumor growth by altering lipid metabolism and triggering cell death

**DOI:** 10.1038/s41419-018-0731-6

**Published:** 2018-06-07

**Authors:** Ge Liu, Kai Wang, Shan Kuang, Ruobing Cao, Li Bao, Rui Liu, Hongwei Liu, Chaomin Sun

**Affiliations:** 1 CAS Key Laboratory of Experimental Marine Biology, Institute of Oceanology, Chinese Academy of Sciences, Qingdao, 266071 China; 2 Laboratory for Marine Biology and Biotechnology, Qingdao National Laboratory for Marine Science and Technology, Qingdao, 266071 China; 3 University of Chinese Academy of Sciences, Beijing, 100049 China; 4 State Key Laboratory of Mycology, Institute of Microbiology, Chinese Academy of Sciences, Beijing, 100101 China

## Abstract

Cancer cells rewire their metabolism to satisfy the demands of uncontrolled proliferation and survival. The reprogramming of lipid metabolism supports tumor growth, metastasis, and therapy-resistance. Therefore, targeting lipid metabolic reprogramming is a potential cancer treatment strategy. We recently isolated the novel natural triterpene GL22 from *Ganoderma leucocontextum*, a traditional Chinese medicine. Here, we show that GL22 significantly inhibits the growth of the liver cancer cell line Huh7.5 in vitro and of Huh7.5-derived tumor xenografts in vivo. We further find that GL22 induces mitochondrial dysfunction and cell death in Huh7.5 cells, in part due to fatty acid immobilization and loss of the mitochondrial lipid cardiolipin, which has vital structural and metabolic functions. Importantly, we demonstrate that GL22 treatment decreases the expression of fatty acid-binding proteins (FABPs), which likely underlies the loss of cardiolipin, mitochondrial dysfunction, and cell death. The over-expressions of FABPs prevented the GL22-induced cell death, loss of cardiolipin, decrease of ATP production, and reduction of oxygen consumption rate in Huh7.5 cells. Our results support targeting lipid metabolism via manipulating FABPs as a cancer treatment strategy, and promote Chinese medicine as an important source of novel anticancer drugs.

## Introduction

Despite recent improvements in treatment strategies, cancer remains one of the leading causes of death worldwide. For instance, patients with hepatocellular carcinoma (HCC) have a 5-year survival rate around only 30–40%^1^. Because HCC is often diagnosed at an advanced stage, many patients are not eligible for surgical resection. Unfortunately, alternative therapies do not substantially improve the prognosis of these patients. Thus, it is of utmost importance to identify new drugs that modulate tumorigenesis and improve patient outcome.

Cancer is characterized by uncontrolled, rapid tumor cell growth. This growth requires an enormous surge in the production of building blocks, including nucleic acids, fatty acids and amino acids. Cancer cells modify their metabolism to meet the increased bioenergetic and biosynthetic requirements. Reprogramming of the lipid metabolic pathway is one of the most significant alterations in tumor cells^2^. Fatty acids (FAs) serve as building blocks for phospholipids within biological membranes^3^. The synthesis of phosphatidylcholine and phosphatidylethanolamine, the major phospholipids found in cellular membranes, is increased in tumors from several tissues and correlates with poor prognosis^4,5^, suggesting that the lipogenic pathway may be a promising target for cancer therapy.

Lipid metabolism is regulated by many processes, including fatty acid transport, synthesis, and oxidation. Fatty acid-binding proteins (FABPs) act as intracellular fatty acid transporters. FABPs coordinate lipid responses in cells and are strongly linked to metabolic and inflammatory pathways^6^. The expression of FABPs is upregulated in many tumors, such as liver, lung, gastric, and ovarian cancers, whereas their expression in peritumoural adipose tissue is downregulated^7^. Recently, the FABPs were shown to be central to lipid-mediated processes and related metabolic pathways, indicating their potential as therapeutic targets for cancer^6^.

Nature has been a source of medicinal products for the treatment of a wide spectrum of diseases, including cancer^8^. Many effective drugs developed from natural organisms have been reported to display anticancer activity through affecting the lipid-related metabolism pathway. For example, betulinic acid (BetA), a cytotoxic plant-derived compound, was reported to induce cancer cell death through cardiolipin modification^9^. Arctigenin, a major compound of *Arctium lappa*, was reported to suppress adipogenesis and activate apoptosis in breast cancer cells^10^.

Traditional Chinese medicine is popular in Chinese and East Asian societies, and plays increasing roles in the modern healthcare system for the development of novel anticancer drugs^11^. The *Ganoderma* species of mushroom is a well-known elixir in China, widely used to treat and prevent various diseases, including cancer^12,13^. We previously isolated new lanostane triterpenes from the cultivated fruiting bodies of *G.leucocontextum*^14^, including GL22, ganoleuconin O. GL22 inhibits HMG-CoA reductase and α-glucosidase in vitro and exhibits cytotoxicity against K562 and PC-3 cancer cell lines^14^. Triterpenes are pharmacologically active compounds that contribute to the antitumor efficacy of *Ganoderma*^14^.

Here, we show that GL22 treatment potently inhibits liver cancer growth in vitro and in vivo by suppressing the expression of FABPs, which leads to FA immobilization and loss of cardiolipin, mitochondrial dysfunction and cell death. Our results provide insight into the potential pharmacological application of GL22 as a novel antitumor agent and support the role of FABPs as important targets in cancer therapy.

## Results

### GL22 inhibits the growth of Huh7.5 cancer cells in vitro and in vivo

**Fig. 1:**
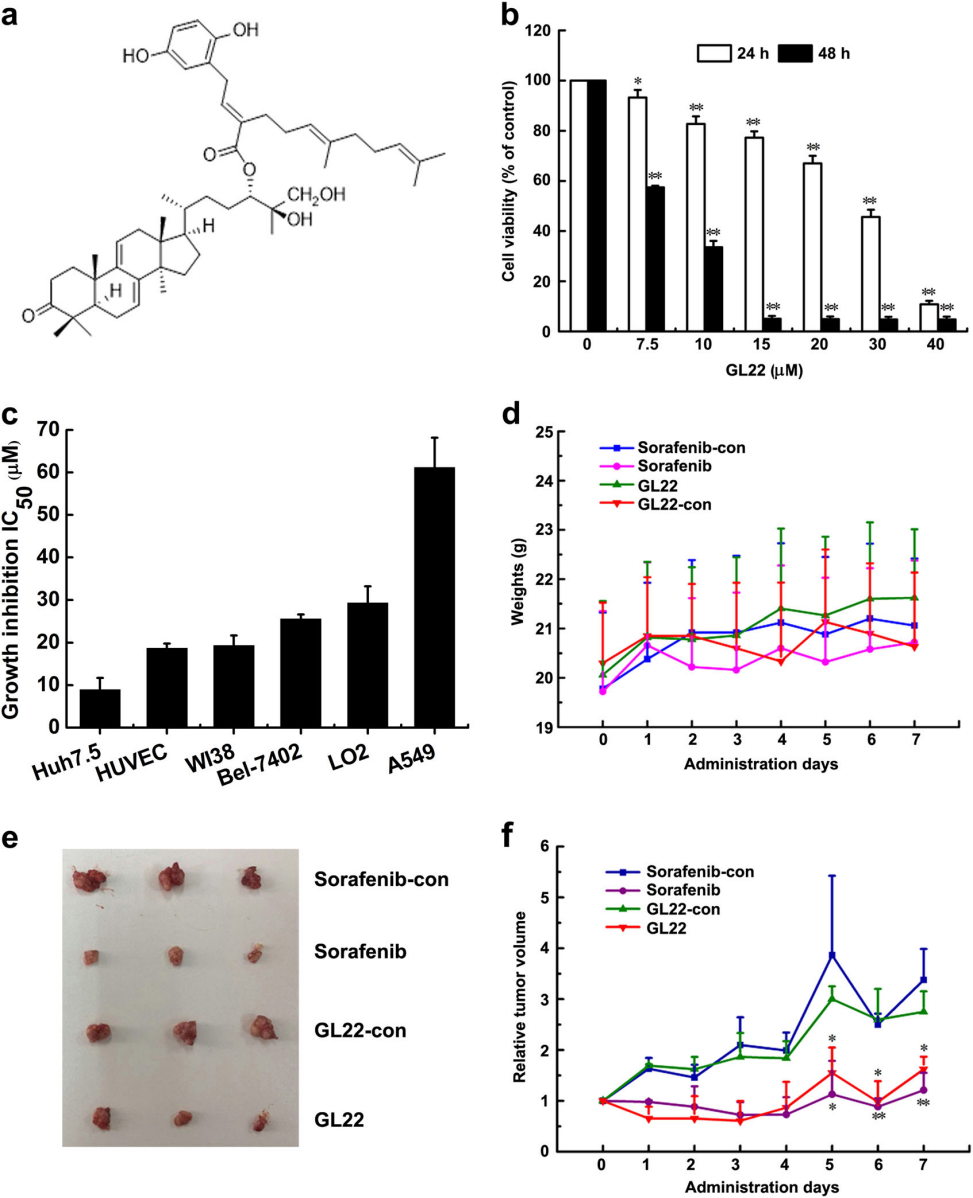
GL22 inhibits Huh7.5 cell xenograft tumor growth in BALB/c-nu mice. **a** Chemical structure of GL22. **b** GL22 inhibited the growth of Huh7.5 cells in a time-dependent and dose-dependent manner (24 and 48 h). **c** After 48 h of treatment with GL22, cell growth was determined by MTT assay, and growth inhibition IC_50_ values were calculated. Values represent the means ± SD of triplicate measurements. **d** GL22 had no effect on the body weights of treated mice. **e** Representative images of the Huh7.5 xenograft tumors from each group at day 7. Sorafenib (30 mg kg^−1^ d^−1^, gavage administration) and GL22 (50 mg kg^−1^ d^−1^, intraperitoneal injection) were used for treatment groups. Control groups were treated with the corresponding solvents same as Sorafenib and GL22 groups. **f** The relative tumor volume (RTV) of each group. *P < 0.05, **P < 0.01 vs. control

To further evaluate the bioactivity of GL22, a triterpene-farnesyl hydroquinone hybrid isolated from *G. leucocontextum* (Fig. 1a), we determined if it would affect the growth of the liver cancer cell line Huh7.5. Indeed, GL22 treatment significantly inhibited the growth of Huh7.5 cells in a time-dependent and dose-dependent manner (Fig. 1b). Next, we evaluated whether GL22 inhibits the growth of various other human cancer and normal cell lines. Of all the cell lines tested, we found that GL22 displayed the most potent growth-inhibitory activity against Huh7.5 cells, with an IC_50_ value of 8.9 μM (Fig. 1c).

We further evaluated the therapeutic potential of GL22 on Huh7.5 cell xenografts in BALB/c-nu mice. The result showed that all mice maintained a normal body weight throughout the treatment (Fig. 1d). GL22 and Sorafenib (positive control) strikingly inhibited Huh7.5 xenograft tumor growth in mice, as determined by tumor volumes (Fig. 1e, f, and Supplementary Fig. 1a). Haematoxylin and eosin (H&E) staining of sections revealed that tumors treated with Sorafenib and GL22 displayed enlarged intercellular spaces and decreased cell density relative to controls (Supplementary Fig. 1b). Thus, GL22 displays antitumor activity in vivo.

### GL22 triggers mitochondrial dysfunction in Huh7.5 cells

**Fig. 2:**
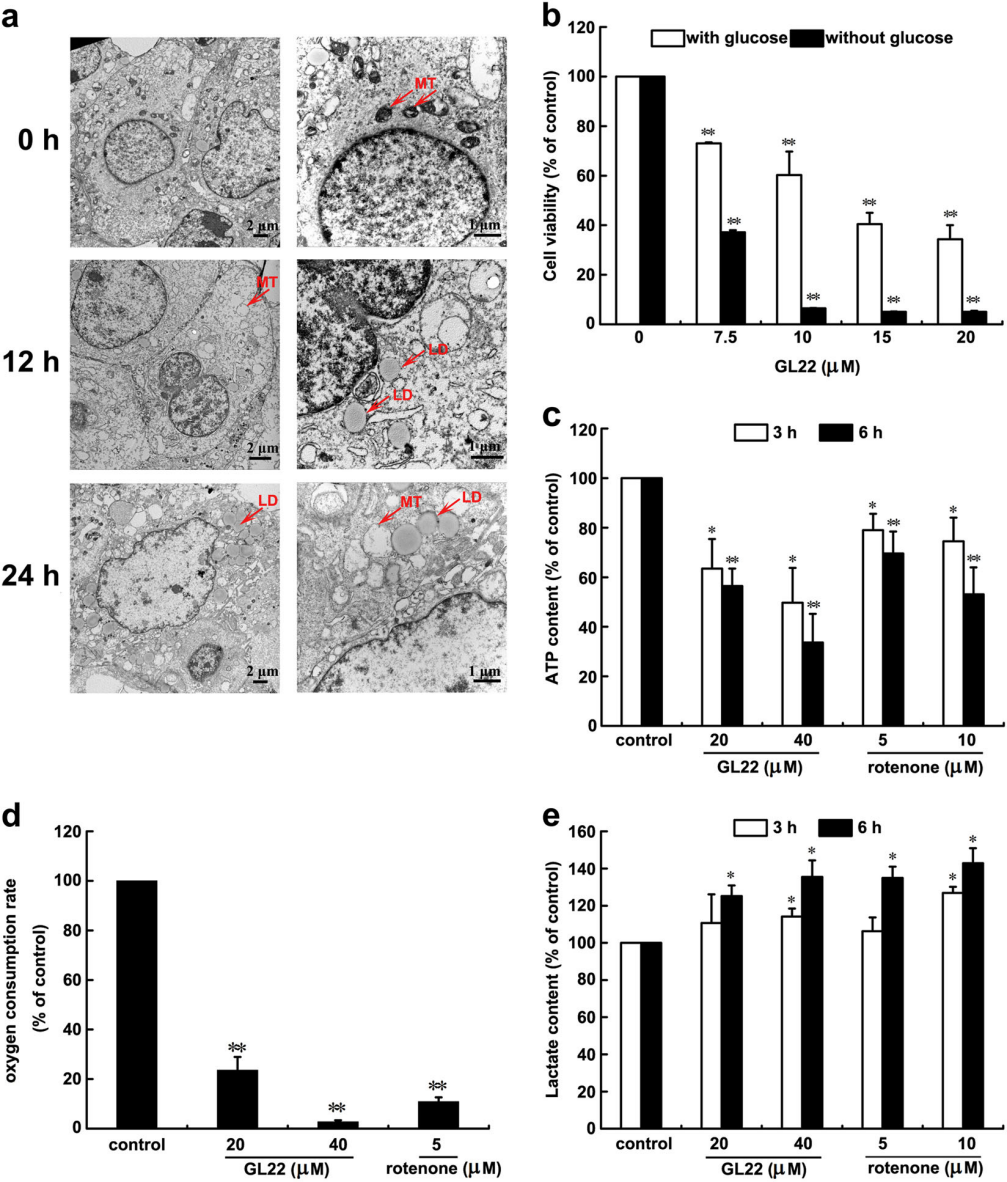
GL22 triggers mitochondrial dysfunction in Huh7.5 cells. **a** GL22 caused primary changes of mitochondrial morphology in Huh7.5 cells. Huh7.5 cells treated with 25 μM GL22 for 0, 12, and 24 h were harvested, stained, and photographed by transmission electron microscopy. Right panels are high-power images for left panels. MT mitochondria, LD lipid drops. **b** Effects of GL22 on Huh7.5 proliferation with or without glucose (36 h). **c**–**e** GL22 treatment of Huh7.5 cells inhibited ATP production, inhibited O_2_ respiration rate and increased lactate production. Values represent the means ± SD of triplicate measurements. *P < 0.05, **P < 0.01 vs. control

To observe the ultrastructural morphology of Huh7.5 cells treated with GL22, we performed transmission electron microscopy (TEM). At the beginning of treatment (*t* = 0 h), Huh7.5 cells had small, morphologically normal, round, or oval mitochondria with a regular distribution of cristae (Fig. 2a, 0 h), whereas cells treated with GL22 for 12 and 24 h displayed altered mitochondrial shape and size, as well as fragmentation of mitochondrial cristae (Fig. 2a, 12 and 24 h). Cells treated with GL22 also displayed a time-dependent increase in the average number and size of lipid droplets (LDs) (Fig. 2a, 12 and 24 h).

The GL22-dependent changes in mitochondrial morphology implied that GL22 inhibits Huh7.5 cell growth in part by abrogating mitochondrial function. A previous study indicated that cancer cells were more sensitive to mitochondrial dysfunction after glucose deprivation^15,16^. We found that the IC_50_ value of GL22 for Huh7.5 cell growth inhibition was lower in glucose-free medium than that in glucose-containing medium (Fig. 2b), suggesting that mitochondrial dysfunction might partly underlie the growth-inhibitory effect of GL22 on Huh7.5 cells.

The primary function of mitochondria is the synthesis of ATP by oxidative phosphorylation. Thus, we measured ATP production as an indicator of mitochondrial function. GL22 treatment resulted in a dose-dependent and time-dependent decrease in the ATP production of Huh7.5 cells, similar to that of rotenone, an inhibitor of the mitochondrial electron transport chain (Fig. 2c). Cellular respiration is closely related to oxidative phosphorylation, and we observed a dose-dependent inhibition of aerobic respiration in Huh7.5 cells treated with GL22 and rotenone (Fig. 2d). In addition, we found that treatment with either rotenone or GL22 increased lactate production of Huh7.5 cells in a concentration-dependent and time-dependent manner, suggesting that an elevation of anaerobic respiration might compensate for the reduced aerobic respiration (Fig. 2e).

In addition, we measured the difference of cell viability between wild type and KO of BAX/BAK in MEF (mouse embryonic fibroblasts). As shown in Supplementary Fig. 2, GL22 displayed more toxic effects on wild type-MEF cells than KO of BAX/BAK-MEF cells after cells were treated with GL22 for 24 (Supplementary Fig. 2a) and 48 h (Supplementary Fig. 2b), respectively. These results suggested that mitochondria were indeed involved in GL22-triggered cell death. The content of cytochrome c in the cytosol was increased (Supplementary Fig. 3a) but decreased in the mitochondria (Supplementary Fig. 3b) of GL22-treated Huh7.5 cells, indicating that GL22 could induce the cytochrome c release from mitochondria to cytosol in Huh7.5 cells and further suggesting that mitochondrial dysfunction and membrane integrity damage were induced by GL22 treatment.

### GL22-mediated growth inhibition arises from loss of cardiolipin

**Fig. 3:**
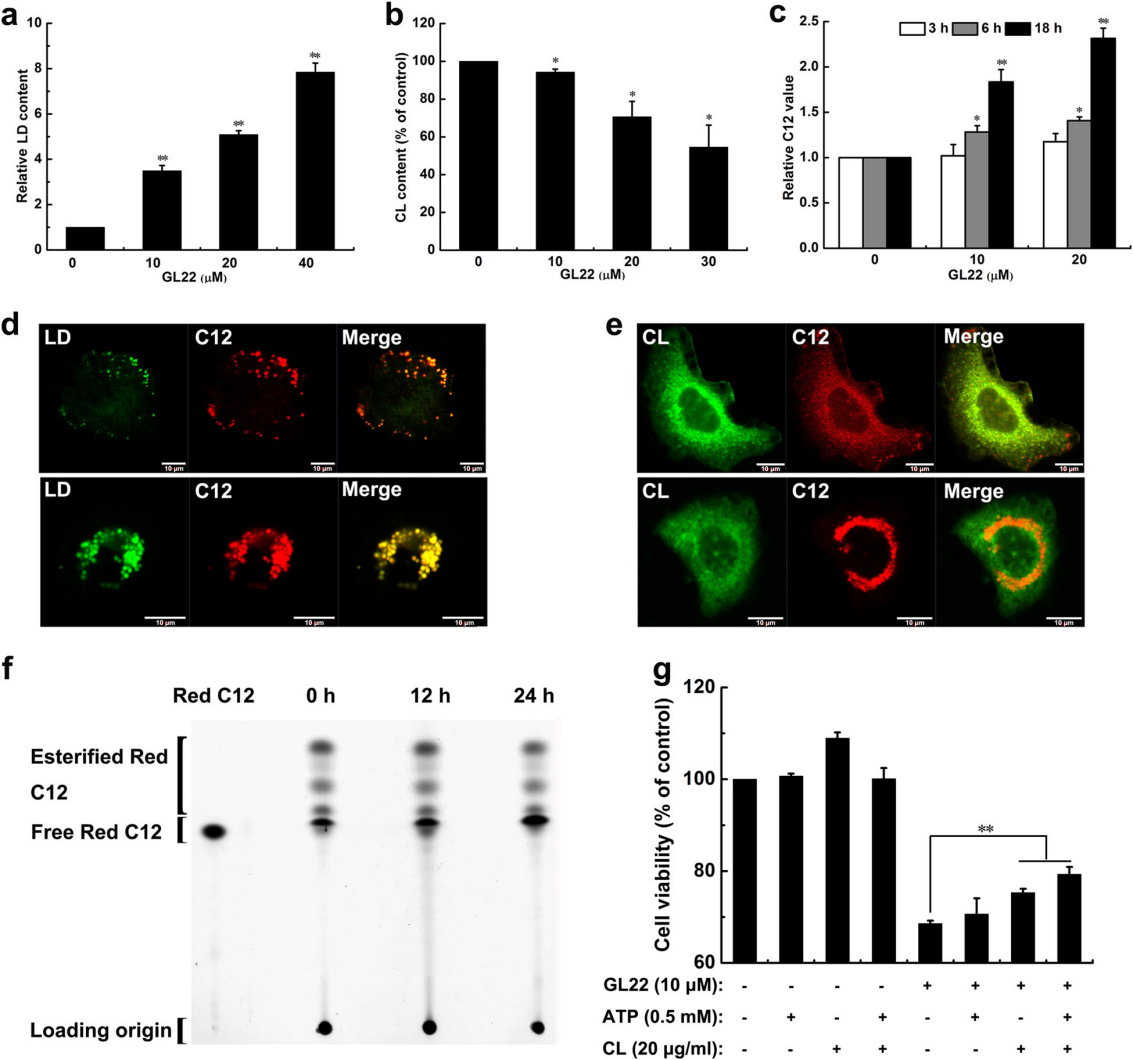
Effects of GL22 on lipid droplets (LDs), cardiolipin, and C12 metabolism in Huh7.5 cells. **a**, **b** GL22 treatment for 24 h increased LD content and decreased cardiolipin content in Huh7.5 cells. After incubated with Bodipy 493/503 fluorescent probe and NAO to label LD and cardiolipin, respectively, cells were harvested and measured by flow cytometry. **c** The metabolism of the fatty acid analog BODIPY C12 was inhibited in Huh7.5 cells treated with GL22 for 3, 6, and 18 h, respectively. **d** Co-localization of LD and C12 in Huh7.5 cells using confocal laser scanning microscopy after cells were treated without (the upper panel) or with 20 μM GL22 (the lower panel) for 24 h. **e** Co-localization of cardiolipin and C12 in Huh7.5 cells using confocal laser scanning microscopy after cells were treated without (the upper panel) or with 20 μM GL22 (the lower panel) for 24 h. **f** TLC resolving Red C12 isolated from Huh7.5 cells treated with 20 μM GL22 for 0, 12, and 24 h, respectively. **g** Cell viability of Huh7.5 cells treated with 10 μM GL22 in the absence or presence of 0.5 mM ATP or 20 μg/mL cardiolipin, as indicated. CL Cardiolipin

Mitochondrial dysfunction and alterations in mitochondrial morphology often accompany lipid-associated diseases and imbalances of cellular lipid homeostasis^17^. A sharp increase in the size and number of LDs were observed in GL22-treated Huh7.5 cells by TEM (Fig. 2a) and the increase of LD content was displayed in a dose-dependent manner as assessed by flow cytometry (Fig. 3a), and also by confocal laser scanning microscopy (Fig. 3d, the upper and lower panels, see below).

LDs store the cellular surplus of lipid molecules^18^, and they are also a repository for the building blocks of membrane phospholipids^19^. Cardiolipin is a signature phospholipid of the mitochondria, where it is synthesized from glycerol-3-phosphate and FAs. We found that cardiolipin content dropped significantly in a concentration-dependent manner after GL22 treatment, correlating with the increased LD content (Fig. 3b).

To further clarify the underling mechanism, we utilized BODIPY 558/568 C12 (Red C12), a saturated FA analog with a tail composed of 12 carbons and a BODIPY 558/568 fluorophore covalently bound at the hydrophobic end. Red C12 has an overall length approximately equivalent to that of an 18-carbon FA^20^. We incubated Huh7.5 cells overnight with Red C12 so it would incorporate into cellular lipids. Cells treated with GL22 showed a time-dependent and dose-dependent increase in Red C12 signal measured by flow cytometry (Fig. 3c), and visualized by confocal laser scanning microscopy (Fig. 3d, the upper and lower panels). The increased Red C12 signal in GL22-treated cells implied a repression of free FA metabolism and mobilization, and an increased storage of excess FAs in LDs to prevent cytotoxicity^21^. These data are consistent with an impaired synthesis of lipids, such as phospholipids and cardiolipin, due to the immobilization of free FAs.

We observed increased co-localization between LDs and Red C12 in GL22-treated cells, further indicating that the accumulation of LDs was closely related to the increase of Red C12 or free FAs (Fig. 3d, the upper and lower panels). On the other hand, cardiolipin and Red C12 showed reduced co-localization in GL22-treated cells compared to controls (Fig. 3e, the upper and lower panels), suggesting that GL22 treatment inhibited cardiolipin biosynthesis from Red C12 building blocks. Collectively, these data suggest that the immobilization of free FAs induced by GL22 treatment triggered an increase in LDs and a decrease in cardiolipin.

Red C12 migrates with slower mobility than its esterified forms due to the difference in polarity. The results of thin-layer chromatography showed that the amount of esterified Red C12 in cells decreased with time after GL22 treatment (Fig. 3f and Supplementary Fig. 4), whereas the amount of free Red C12 increased, consistent with GL22-dependent inhibition of free FA transport and mobilization.

The addition of ATP did not significantly improve the growth of GL22-treated cells (Fig. 3g), suggesting that the growth inhibition induced by GL22 does not rely exclusively on ATP depletion. However, exogenously added cardiolipin, as well as co-addition of ATP and cardiolipin, partly restored cell growth in the presence of GL22 (Fig. 3g). Thus, GL22-induced abnormalities in cardiolipin content appear to underlie, at least in part, the growth inhibition mediated by GL22 treatment.

### GL22 induces apoptosis in Huh7.5 cells

**Fig. 4:**
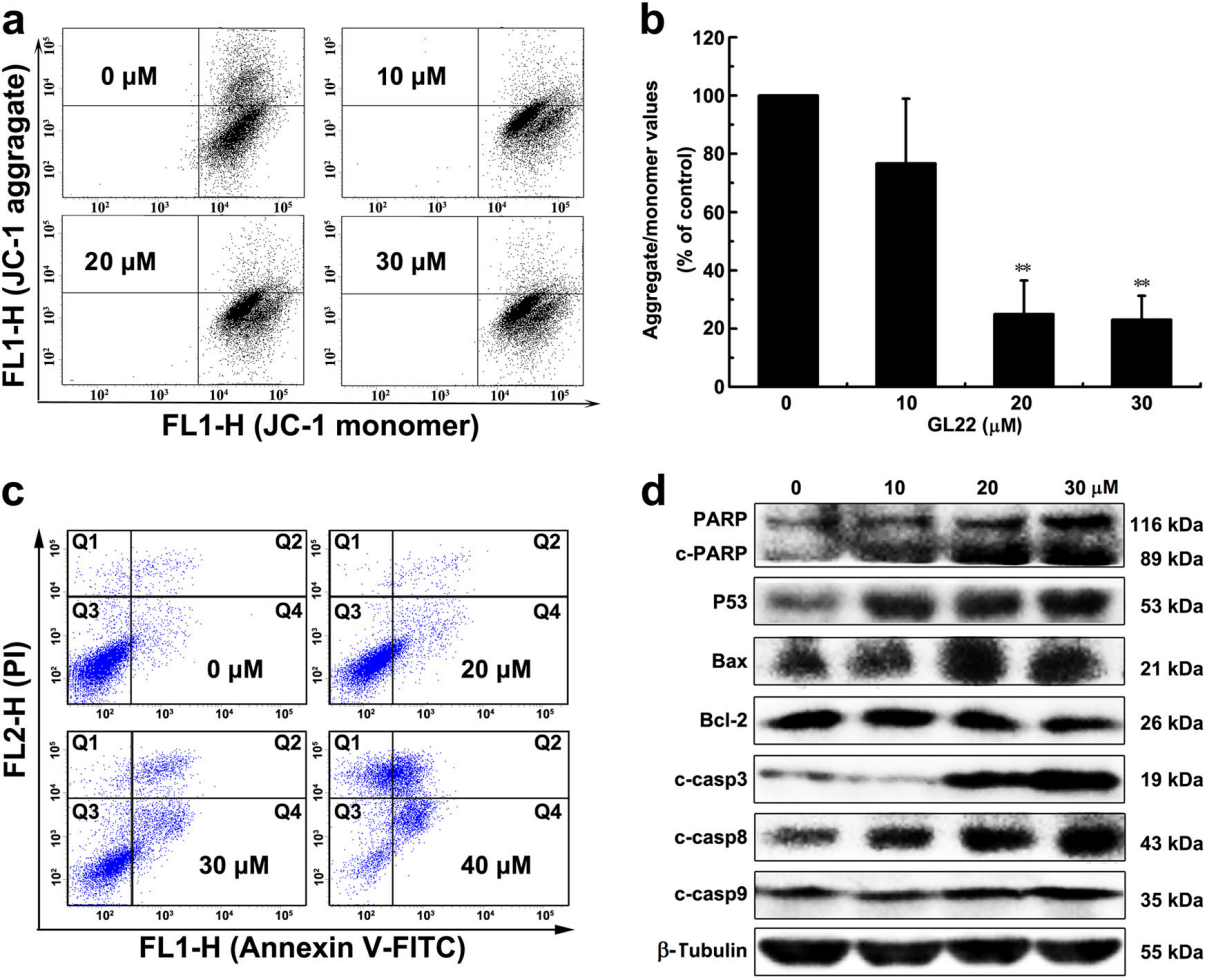
Effects of GL22 on the death of Huh7.5 cells. **a**–**c** GL22 decreased mitochondrial membrane potential (MMP) and induced apoptosis in Huh7.5 cells after cells were treated without or with various concentrations of GL22 for 12 and 24 h, respectively. **d** The apoptosis-related proteins expression induced by GL22 in Huh7.5 cells

Cardiolipin is emerging as an important regulator of several of the steps in cell death^22^. Considering that GL22 treatment of Huh7.5 cells significantly decreased the amount of cardiolipin in mitochondria, we next asked whether it induced apoptosis. The dissipation of the mitochondrial membrane potential (MMP), activated by multiple stress signals, is recognized as an irreversible step in the death cascade^23^. We found that MMP is disrupted in GL22-treated cells (Fig. 4a, b).The induction of apoptosis were strikingly observed, with a dose-dependent increase in the frequency of early and late apoptotic cells after treatment with GL22 (Fig. 4c and Supplementary Fig. 5a).

Apoptosis is a strictly regulated process. The tumor suppressor protein P53 works as both an inducer and regulator of apoptosis^24^. Bcl-2 family proteins are also important mediators of apoptosis^25^ and the ratio of Bax/Bcl-2 is a major checkpoint in the intrinsic apoptosis pathway. GL22 treatment significantly upregulated the levels of P53 and Bax, and downregulated the level of Bcl-2, resulting in an increase in the ratio of Bax/Bcl-2 in Huh7.5 cells (Fig. 4d and Supplementary Fig. 5b). There are two distinct pathways that initiate apoptosis, designated as the mitochondrial and death receptor pathways^26^. The mitochondrial pathway can be activated by diverse stress signals, such as toxins, reactive oxygen species, and genotoxic stress, resulting in collapse of MMP. Collapse of MMP causes the release of cytochrome c and auto-activation of caspase-9, eventually triggering apoptosis. Death receptor-mediated pathways is the interaction of the cell surface receptors with their ligands to activate the downstream effectors (caspase-8), finally leading to apoptosis. The hallmarks of the mitochondrial-mediated intrinsic apoptotic pathways, caspases 3, 9, and PARP cleavage, were activated by GL22 treatment (Fig.4d and Supplementary Fig. 5b). Cleaved-caspase-8 protein level was also increased in the GL22-treated Huh7.5 cells, consistent with death receptor-mediated extrinsic apoptotic pathways (Fig. 4d and Supplementary Fig. 5b). Thus, it appears that both the mitochondrial-mediated intrinsic and death receptor-mediated extrinsic apoptotic pathways are involved in GL22-induced apoptosis of Huh7.5 cells.

### GL22 reduces the expression of FABPs in vitro and in vivo

Next, we performed a proteomic analysis and identified 128 and 141 proteins that were differently expressed (1.3-fold change cutoff and *P* < 0.05) (Supplementary Tables 1, 2 and 3) in Huh7.5 cells treated with GL22 for 12 and 24 h, respectively. Among these differently expressed proteins, 12 proteins were involved in fatty acid metabolism and downregulated after GL22 treatment (Supplementary Fig. 6). These data are consistent with our findings above, and with emerging evidence that GL22 may affect lipid metabolism.

**Fig. 5:**
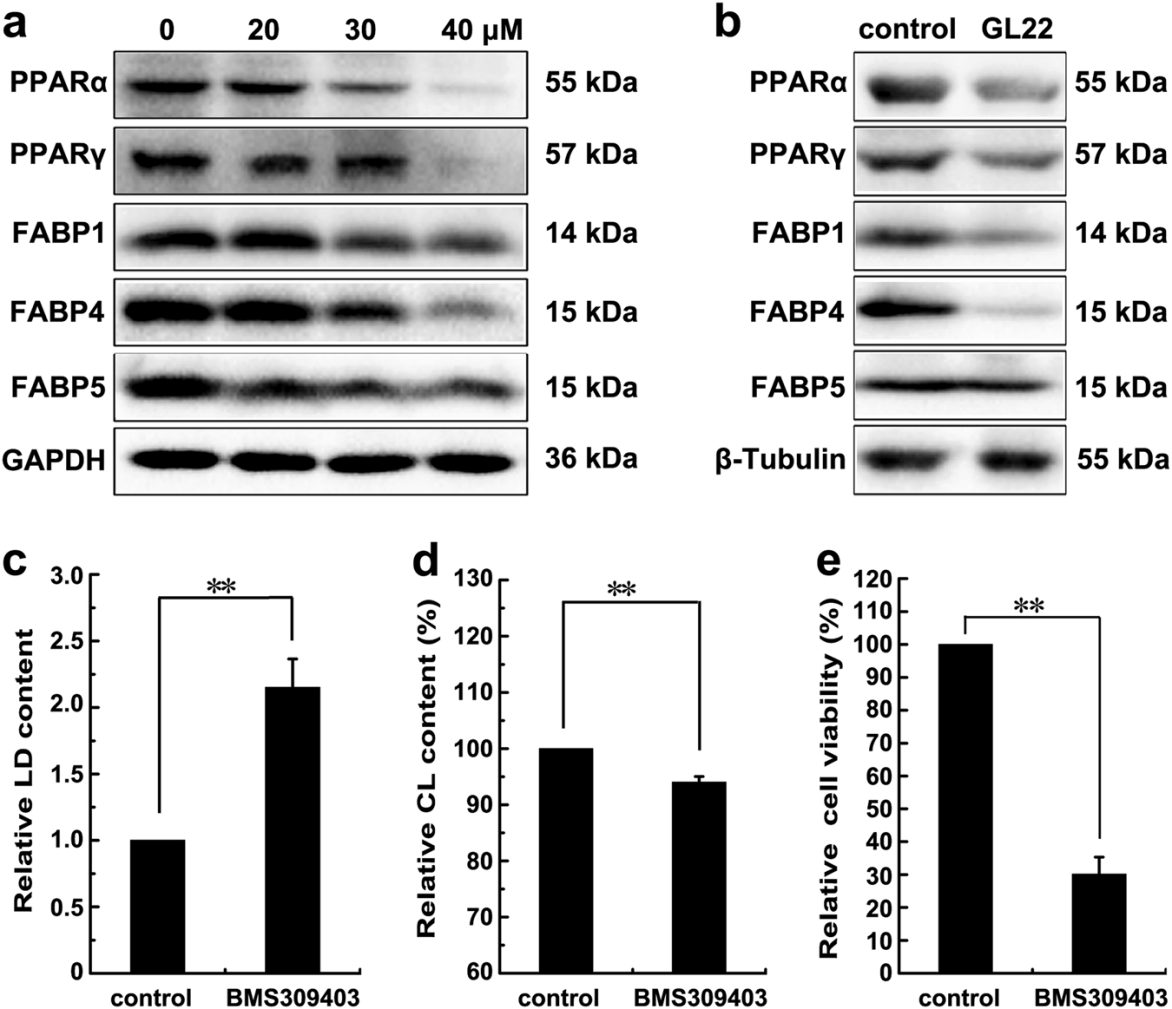
GL22 inhibits the expression of FABPs. **a** The expression of FABPs (FABP1, 4, 5) decreased in Huh7.5 cells treated with GL22 for 24 h. **b** GL22 inhibited the expression of FABPs (FABP1/4/5) in xenografts. Xenograft animals were administrated GL22 at dose of 50 mg/kg, whereas control animals received equivalent volumes of solvent. GL22 treatment was initiated when tumors were palpable and continued for 1 week. **c**–**e** BMS309403 treatment caused LD accumulation, cardiolipin content reduction and cell viability disease in Huh7.5 cells after cells were pre-treated for 10 min with 50 μM BMS309403, which partically phenocopy the effect of GL22. After treatment, LD and cardiolipin content were measured using flow cytometry as described in Fig. 3. Data of three independent experiments are shown. *P < 0.05, **P < 0.01 vs. control

FABPs reversibly bind FAs with high affinity. The FABP content in most cells is generally proportional to the rate of fatty acid metabolism^27^. GL22 treatment significantly decreased the levels of multiple FABPs (FABP1/4/5) (Fig. 5a), consistent with the results of the proteomic analysis (Supplementary Fig. 6). We also determined the levels of the peroxisome proliferator-activated receptor components PPARα and PPARγ, which play a crucial role in lipid metabolism through transcriptional regulation of genes, including FABPs. The levels of PPARα and PPARγ were also significantly decreased after GL22 treatment (Fig. 5a), further confirming the inhibitory effect of GL22 on the PPAR–FABPs signaling pathway.

The mRNA levels of FABP4, PPARα, and PPARγ were decreased in GL22-treated Huh7.5 cells (Supplementary Fig. 7). However, the transcriptional levels of FABP1 and FABP5 were increased after GL22 treatment. Discrepancies between RNA and protein could be explained by post-transcriptional regulatory mechanisms and small non-coding RNAs.

Moreover, the expression levels of PPARα, PPARγ, FABP1, FABP4, and FABP5 were downregulated in GL22-treated xenograft tumors (Fig. 5b), indicating that GL22 exerted a significant anticancer effect against liver cancer in vivo mediated by PPAR–FABPs signaling pathway.

More importantly, the accumulation of LDs induced by GL22 remarkably decreased in a dose-dependent manner in Huh7.5 cells pre-treated with clofibrate (a PPAR agonist) (Supplementary Fig. 8a), suggesting that there was a relationship between LD boost and PPAR. Moreover, pre-treatment with clofibrate significantly rescued the growth-inhibitory effects and the reduced expression levels of FABP1/4/5 induced by GL22 (Supplementary Figs. 8b, c). Therefore, GL22 could directly affect PPAR then lead to downregulation of FABPs and decreased cell viability.

### The FABPs inhibitor, BMS309403, can partially recapitulate the effect of GL22 on Huh7.5 cells

Emerging evidence suggests that FABPs are associated with the transfer of FAs from LDs to mitochondria^6,20^. We treated Huh7.5 cells with BMS309403, a FABPs (FABP1, 3–5, and −7) inhibitor^11,28^, to identify if it could generate a similar effect to that seen in the GL22-treated cells. Consistent with those observed after GL22 treatment, BMS309403 also caused the drop of cell viability, the increase of LDs and the decrease of cardiolipin in Huh7.5 cells (Fig. 5c–e).

### The over-expressions of FABPs (FABP1/4/5) rescue GL22-induced the decrease of cell viability, increase of LDs, decrease of cardiolipin, drop of ATP production, and reduction of oxygen consumption rate

We measured the difference in expression profiles of FABPs (FABP1/4/5) among six human cell lines, including human liver cancer cell line (Huh7.5), human lung cancer cell line (A549), human umbilical vein endothelial cell (HUVEC), normal human fetal lung fibroblast (WI38), human liver cancer cell line (Bel-7402), and normal human liver cells (LO2). The result showed that the expression levels of FABPs (FABP1/4/5) in Huh7.5 cells were the highest among these six cell lines (Supplementary Fig. 9), corresponding to the most sensitive to GL22 treatment among these six cell lines (Fig. 1c). The data confirmed that the difference in the expression levels of FABPs in various cells may explain the difference in their sensitivities to GL22 treatment to a certain extent.

**Fig. 6:**
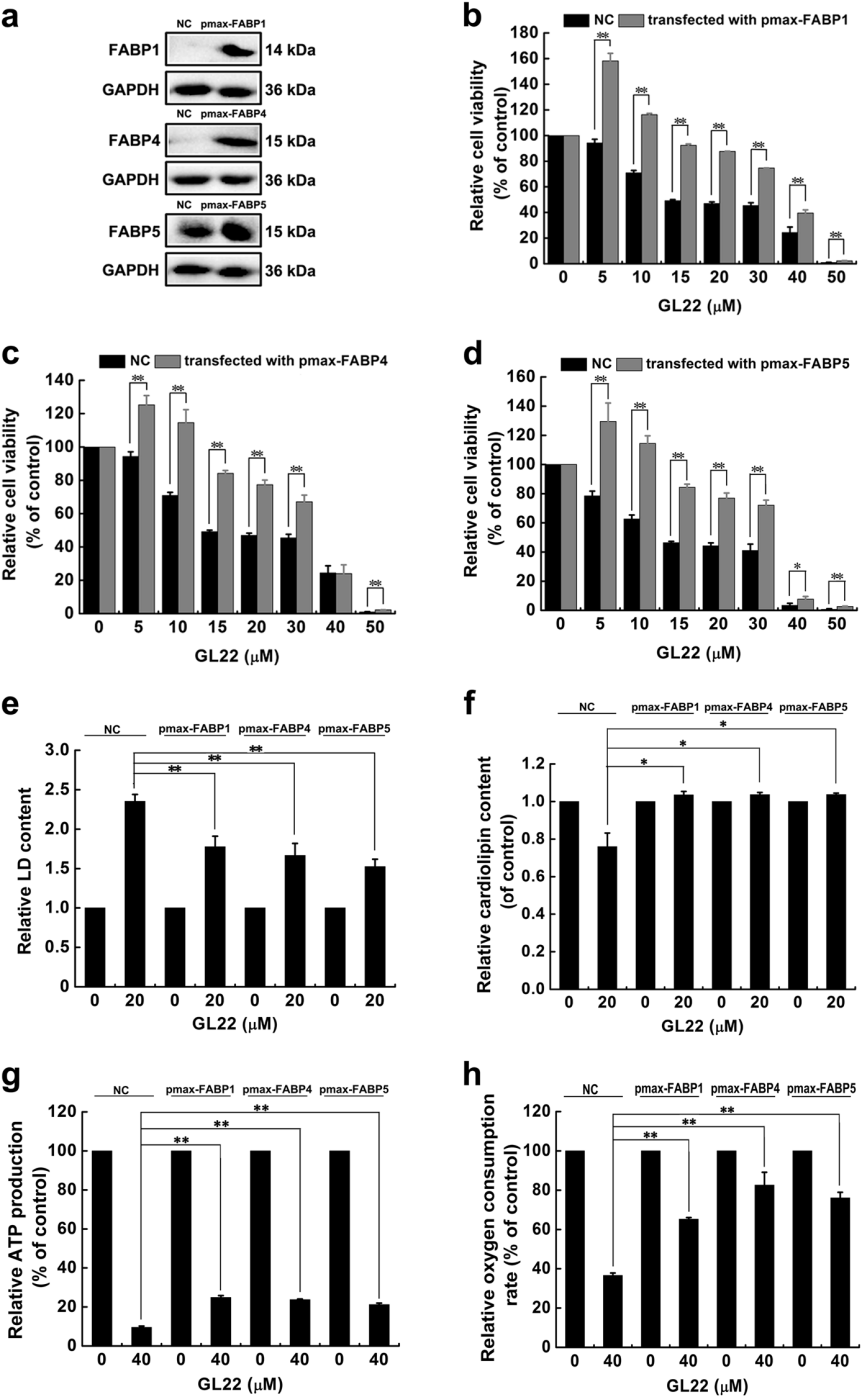
The over-expressions of FABPs (FABP1/4/5) in the Huh7.5 cells rescued GL22-induced the decrease of cell viability, increase of LD, decrease of cardiolipin, drop of ATP production, and reduction of oxygen consumption rate. **a** The expression levels of FABPs (FABP1/4/5) were detected by western blotting after Huh7.5 cells were transfected with pmax-FABP1, pmax-FABP4, and pmax-FABP5 for 48 h. **b**–**d** The over-expressions of FABP1, FABP4, and FABP5 in the Huh7.5 cells rescued the GL22-induced the reduction of cell viability. **e**–**h** The over-expressions of FABP1, FABP4, and FABP5 in the Huh7.5 cells rescued the GL22-induced the accumulation of LD, the loss of cardiolipin, the decrease of ATP production, and the reduction of oxygen consumption rate. Data of three independent experiments are shown. *P < 0.05, **P < 0.01 vs. control

To further evaluate the functional importance of the GL22-mediated inhibition of FABPs, FABPs (FABP1/4/5) were cloned by PCR using primers (Supplementary Table 4) and over-expressed in Huh7.5 cells through transfection. As shown in Fig. 6a, the expression levels of FABPs (FABP1/4/5) in Huh7.5 cells were dramatically increased after transfection for 48 h. The decreased cell viability, accumulation of LDs and decrease of cardiolipin in GL22-treated Huh7.5 cells were all restored by over-expressions of FABPs (FABP1/4/5) (Fig. 6b–f). Moreover, over-expressions of FABPs also prevented the ATP production and oxygen consumption rate-indicators of mitochondrial function from declining induced by GL22 (Fig. 6g, h). These results suggested that the reduced expression of FABPs lead to the immobilization of FAs, the accumulation of LDs, the decrease of cardiolipin, mitochondrial dysfunction, and cell death.

## Discussion

Here, we report that GL22, a triterpene-farnesyl hydroquinone hybrid isolated from the fruiting bodies of *G. leucocontextum*, exerts an anticancer effect in vitro and in vivo by inhibiting FA mobilization and cardiolipin biosynthesis, due to the reduced expression of FABPs. To our knowledge, this is the first report of fungal secondary metabolites stimulating antitumor activity by targeting FABPs and depleting cardiolipins, thereby expanding the medicinal value of *G. leucocontextum*. Our data suggest that the *Ganoderma* species is a promising source of new anticancer agents.

Cancer cells change their metabolism to satisfy the demands of growth and survival. This metabolic reprogramming is considered a hallmark of cancer^29^. In this study, we found that GL22 alters mitochondrial shape and ultrastructure (Fig. 2a), triggering mitochondrial dysfunction, including reduced ATP production (Fig. 2c), decreased aerobic respiration (Fig. 2d), and increased compensatory anaerobic respiration (Fig. 2e). These GL22-induced defects in mitochondrial structural integrity and function likely arise, in part, from the effects of GL22 on cellular lipid homeostasis^17^.

Accumulating evidence suggests that cancer cells show alterations in different aspects of lipid metabolism, which could affect numerous important cellular processes, including cell growth, proliferation, differentiation, and survival^30^. Medes et al. first demonstrated that FA synthesis occurs at very high rates in tumors^31^, suggesting that lipid metabolism, in particular FA metabolism, is tightly linked to cancer cell growth and proliferation^32^. Lipids are synthesized from FAs and serve as important building blocks of biological membranes. A critical limitation in lipid biosynthesis is the availability of free FAs. If cellular FA flow is blocked, free FAs would accumulate within LDs to maintain the balance of cellular lipid levels^20,33^, as we observed upon GL22 treatment (Fig. 3a). We found that GL22 treatment of Huh7.5 cells induced an accumulation of the FA analog Red C12 (Fig. 3c), and an increased co-localization between LD and Red C12 (Fig. 3d). These findings suggest that GL22 treatment inhibits the mobilization of free FA.

Given that free FAs are the building blocks of lipids, GL22-mediated immobilization of FAs inevitably results in failure of lipid biosynthesis and in turn, disrupts the generation of biological membranes and cellular functions. Cardiolipin, the signature phospholipid of the mitochondria, has diverse biological functions, including mitochondrial biogenesis^34^, mitochondrial bioenergetics^35^, mitochondrial dynamics^36^, and cell death^22,37–39^. The blocked FA transport induced by GL22 led to the inhibited biosynthesis of cardiolipin (Fig. 3b). Cardiolipin is emerging as an important player in the regulation of several steps in cell death, and the cell death induced by GL22 was partially prevented by exogenously supplied cardiolipin (Fig. 3g). Thus, the decrease of cardiolipin content accounts, in part, for the antitumor activity of GL22.

FABPs are known as intracellular lipid chaperones. They reversibly bind FAs and participate in the cellular FA flow, including import, storage, transport, mobilization, and export^6^. FABPs are over-expressed in some cancer cells and their expression correlates with tumor aggressiveness in patients^7^. We found that GL22 suppressed the expression of FABPs (FABP1/4/5) in Huh7.5 cells (Fig. 5a). BMS309403 is a rationally designed, potent inhibitor of FABPs (FABP1, 3–5, and −7), which interacts with the FA-binding pocket to inhibit the binding of endogenous FAs^11,28^. BMS309403 can partially phenocopy the effect of GL22 on lipid metabolism and cell death in Huh7.5 cells (Fig. 5c–e). Moreover, the over-expressions of FABPs (FABP1/4/5) in Huh7.5 cells prevented the GL22-mediated accumulation of LDs, loss of cardiolipin, drop of ATP production, and reduction of oxygen consumption rate, and even the cell death (Fig. 6b–h), indicating that the inhibition of FABPs expression contribute to the failed FAs transport, decrease of cardiolipin synthesis, and cell death. Targeting FABPs in cancer cells provides a novel mechanism of inducing cell death. It has been reported that FABPs inhibitors could exert anticancer activities through the suppression of tumor metastasis^40^. The discovery of pharmacological agents that modify FABPs function and/or expression may therefore offer a new class of therapeutic agents against cancer. FAs are the main energy resources for the heart, and FA oxidation account for 40–80% of heart energy. The reduced FABPs induced by GL22 inevitably affect the intracellular transport of FAs from cytosol to other compartments, thus possibly causing heart toxicity for lack of FAs supply. Further structural modification and formulation investigation on GL22 will provide solutions to overcome the possible heart toxicity.

**Fig. 7:**
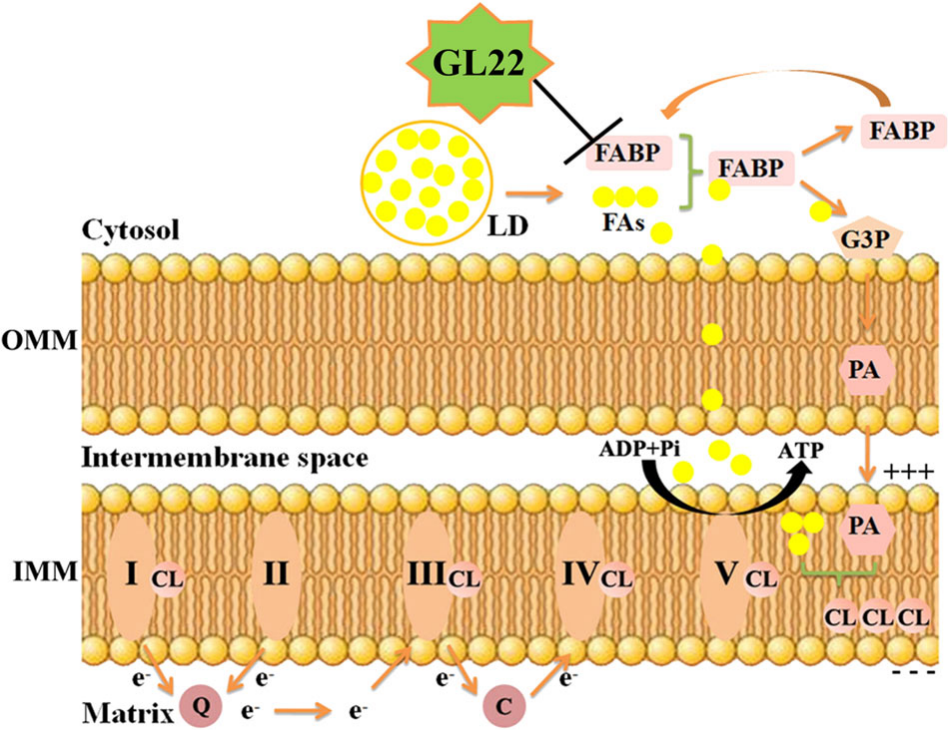
Model of molecular mechanism induced by GL22. GL22 displayed antitumor activity through mitochondrial dysfunction by inhibiting FABPs. Cardiolipin is a unique phospholipid and almost exclusively exists in the IMM, where cardiolipin is needed for aggregating and anchoring the oxidative phosphorylation proteins, including complexes I, III, and IV. Cardiolipin is synthesized in mitochondria from its basic building blocks, G3P and FAs. More specifically, G3P is acylated to PA in the OMM. PA is translocated from OMM to IMM, and is converted to CL by further acylation. In this pathway, FAs are the donors of all fatty acyl groups. *OMM* outer mitochondrial membrane, *IMM* inner mitochondrial membrane, *CL* cardiolipin, *LDs* lipid droplets, *FA* fatty acid, *G3P* glycero-3-phosphate, *PA* phosphatidic acid, *FABP* fatty acid-binding protein, *Q* coenzyme Q, *c* cytochrome c; I, II, III, and IV represent electron transport chain complex I, II, III, and IV

In summary, GL22 displays robust antitumor activity against Huh7.5 cells in vitro and in vivo, supporting the potential medicinal and commercial value of *G. leucocontextum*. GL22 inhibited the expression of FABPs, leading to disrupted FA transport, reduced cardiolipin synthesis, mitochondrial dysfunction, and cell death (Fig. 7). These results reveal GL22 as a new potential cancer therapeutic and further support targeting FABPs in cancer treatment.

## Experimental procedures

### Materials

GL22, isolated from the Tibetan Medicinal Mushroom *Ganoderma leucocontextum*, was provided by Professor Hongwei Liu. GL22 (>98% purity) was dissolved in dimethylsulfoxide at 20 mM and stored at −80 °C. RPMI-1640 was purchased from GIBCO (cat#1786044, Invitrogen, Grand Island, NY, USA). Fetal bovine serum (FBS) was bought from PAN Biotech. (cat#P30-3302, Adenbach, Bavaria, Germany). JC-1 MMP detection assay kit (cat#C2006) and intracellular ATP detection kit (cat#S0026) were supplied by Beyotime Institute of Biotechnology (Shanghai, China). Annexin V-FITC/PI apoptosis detection kit was obtained from Nanjing KeyGEN Biotech. Co., Ltd. (cat#KGA107, Nanjing, Jiangsu, China). The enhanced chemiluminescence (ECL) was provided by Millipore (cat# WBKLS0100, Millipore Corporation, Billerica, MA, USA). The antibodies against P53 (cat#BM0101) and β-tubulin (cat#BM3897) were purchased from Wuhan Boster Biological Technology, Co., Ltd. (Wuhan, Hubei, China). Rabbit antibodies against PPARα (cat#WL00978), PPARγ (cat#WL0269), PARP (cat#WL01932), Bax (cat#WL01637), Bcl-2 (cat#WL01556), cleaved-Caspase-3 (cat#WL01857), and cleaved-Caspase-9 (cat#WL01838) were obtained from Wanleibio (Shenyang, Liaoning, China). Antibodies against GAPDH (cat#60004-1-Ig), FABP1 (cat#13626-1-AP), FABP4 (cat#12802-1-AP), and FABP5 (cat#12348-1-AP) were obtained from Proteintech (Wuhan, Hubei, China). Bodipy 493/503 (cat#D3922) and Bodipy 558/568 C12 (cat#D3835) were purchased from Invitrogen (Eugene, OR, USA). 10-nonyl acridine orange (NAO, cat#N-4002) was the product of US Everbright. BMS309403 was bought from APExBIO (cat#B7794, Houston, TX, USA). Cardiolipin (cat#C1649) was provided by Sigma-Aldrich Co. LLC. All other reagents used in the experiment were analytical grade or higher.

### Cell culture

All the cell lines used were obtained from the American Type Culture Collection. Huh7.5 cells were cultured in RPMI-1640 medium supplemented with 10% FBS, 100 U/mL penicillin, and 100 μg/mL streptomycin. All experiments were carried out with the same batch of Huh7.5 between passages 2–5. Glucose deprivation was achieved by replacing the medium of cells with glucose-deprived medium after rinsing cells with phosphate-buffered saline (PBS). The glucose-deprived medium was glucose-free RPMI-1640 supplemented with 10% FBS.

### Cell proliferation assay

The cytotoxicity of GL22 against Huh7.5 cells was determined by MTT method. Briefly, logarithmically growing Huh7.5 cells (6 × 10^3^) were plated in the 96-well plate at 37 °C for 24 h. Then, cells were treated with the varying concentrations of GL22 (0, 7.5, 10, 15, 20, 30, 40 μM) for 24 and 48 h. MTT solution (5 mg/mL, 20 μL/well) was added and incubated for another 4 h. DMSO was added to each well to dissolve purple crystals of formazan with gentle shaking for 10 min, and OD490 was read by a multi-detection microplate reader (Infinite M1000 Pro, TECAN). Relative cell viability was presented as a percentage relative to the control group. All experiments were performed three times.

### Xenograft tumor mouse model

BALB/C nu-nu male mice, 4 weeks old, were obtained from Beijing Vital River Laboratory Animal Technology Co., Ltd. (Beijing, China). Mice were maintained in cages with free-accessed food and water under a 12 h dark/light cycle and a temperature of 22 ± 3 °C. All studies in mice were approved by IOCAS (Institute of Oceanology, Chinese Academy of Sciences) Laboratory Animal Care and Ethics Committee in accordance with the animal care and use guidelines. A total of 3 × 10^6^ Huh7.5 cells were subcutaneously injected into the right fore flank of each nude mouse. The daily drug treatment began when tumor size reached ~100 mm^3^ and continued for further 1 week as following: GL22 group, GL22 (50 mg kg^−1^ d^−1^, intraperitoneal injection) dissolved in assisted solvent (DMSO/Tween-80/water, 8.3/5/86.7, vol/vol/vol); sorafenib-positive group, Sorafenib (30 mg kg^−1^ d^−1^, gavage administration) in 5% DMSO; Control groups were given with the assisted solvent same as GL22 group and Sorafenib group. Body weight and tumor volumes were measured every day with a balance or with a vernier caliper. The tumor volume was calculated with the formula: 1/2 × [length × (width)^2^], and the relative tumor volume (RTV) was calculated with the formula: *V*_*n*_/*V*_0_ (*V*_*n*_ means the tumor volume at the “*n*” day after administration and *V*_0_ means the tumor volume before administration). After 1 week treatment, animals were sacrificed with decapitation and tumor tissues were collected for further analysis.

### Histology

After 7 days of treatment, the mice were killed by cervical dislocation, and tumors were excised. The tumor tissues were fixed in 4% paraformaldehyde for 12 h. Followed by 5%, 20%, 30% sucrose gradient dehydrated 1 day, until the tumors completely sank to the bottom. Then the tumors were frozen, and processed for OCT-embedded sectioning at 8 μm and stained with H&E.

### Electron microscopy assay

After the indicated treatment, cells were fixed in 2.5% glutaraldehyde and postfixed with 1% osmium tetroxide (OsO_4_) and kaliumhexacyanoferrate (K_3_FE(CN)_6_) in cacodylate buffer. Subsequently, the cells were dehydrated in an alcohol series and embedded into Epon (LX-112 resin Ladd Research, Williston, VT, USA). Ultrathin sections were collected on formvar-coated grids, counterstained with uranil acetate and lead citrate and visualized with transmission electron microscope.

### Measurement of cellular ATP content

Cellular ATP content was measured following the manufacturers’ instructions (intracellular ATP detection kit, Beyotime). Briefly, Huh7.5 cells seeded in 12-well plate were incubated up to 3 or 6 h with GL22 or rotenone (positive control) at the concentrations indicated. Then, cells were rinsed three times with PBS and homogenized in ice-cold ATP lysis buffer. After centrifugation for 10 min at 4 °C, the supernatant of each sample was combined with equal volume of ATP assay buffer and then the level of chemiluminescence was immediately measured using a multi-detection microplate reader (Infinite M1000 Pro, TECAN). The ATP level of cells treated with DMSO in complete medium was considered as 100%.

### Determination of lactate content

Huh7.5 cells were cultured in a 12-well plate and treated for 3 or 6 h with GL22 (20 and 40 μM) or rotenone (5 and 10 μM, positive control) in FBS-free RPMI-1640. Lactate in the medium was measured with a lactate assay kit (cat#A020-1, Nanjing Jiancheng Bioengineering Institute, Nanjing, Jiangsu, China).

### Measurement of extracellular oxygen consumption rate

Changes in extracellular oxygen consumption rate (OCR) of cells were monitored using the Extracellular O_2_ Consumption Assay Kit (cat#ab197243, Abcam) as previously described^41^. Briefly, Huh7.5 cells (6 × 10^4^/well) were plated into 96-well (black wall) clear bottom plates, and incubated in a CO_2_ incubator at 37 °C overnight. Then the culture medium was removed and replaced with 150 μL of fresh culture media supplemented with GL22 (20 and 40 μM) or rotenone (5 μM). An aliquot of 10 μL reconstituted Extracellular O_2_ Consumption reagent and two drops pre-warmed High Sensitivity mineral oil were added into each well, and the plate was immediately monitored using a multi-detection microplate reader (Infinite M1000 Pro, TECAN). Extracellular O_2_ Consumption signal was measured at 1.5 min intervals for 90–120 min at 37 °C using excitation and emission wavelengths of 380 and 650 nm, respectively.

### Lipid extraction and thin layer chromatography assay for fluorescent lipids

To characterize the metabolism of the fluorescent FA analogs in cells treated with GL22, Huh7.5 cells were incubated with 2 μM BODIPY 558/568 C12 (Red C12, Life Technologies) for 16 h in culture medium as describe previously^20^. Cells were then washed three times followed by further incubation with 20 μM GL22 for indicated time (0, 12, or 24 h). After that, cells were washed with PBS and harvested into 1 mL ice-cold PBS and immediately transferred to 4 mL chloroform to extract lipid. The lipid phase was collected and evaporated under N_2_. The lipids were dissolved in chloroform and spotted onto silica gel H thin layer chromatography (TLC) plates. Separation of lipids was performed by developing the plates in a solvent system of heptane/isopropyl ether/acetic acid, 60:40:4 (vol/vol/vol)^42^ and flurorescent lipids were visualized using a Typhoon FLA 9500 Imager (GE Amersham).

### Quantitation of cardiolipin, LDs, and fluorescent 558/568 C12 by flow cytometry analysis

The cellular cardiolipin contents were measured using NAO (10-nonyl acridine orange) as a specific fluorescence dye as described previously^43^. Huh7.5 cells were plated into 6-well plate and treated with GL22 (0, 10, 20, and 40 μM) for 24 h. Cells were then incubated with 5 μM NAO for 30 min and harvested by trypsinization. The fluorescence was determined by flow cytometry (FACS Aria II, BD, San Jose, California, USA).

For the quantitation of LD contents, Huh7.5 cells treated with GL22 (0, 10, 20, and 30 μM) for 12 h were labeled with 10 μg/mL Bodipy 493/503 fluorescent probe for 30 min and then the fluorescence was determined by flow cytometry.

For the measurement of Bodipy 558/568 C12, Huh7.5 cells were first incubated with 2 μM BODIPY 558/568 C12 for 16 h in culture medium as describe before, and then cells were treated with GL22 (0, 10, and 20 μM) for indicated time (3, 6, or 18 h). Cells were harvested and the Red C12 fluorescence signal was analyzed by flow cytometry.

### Confocal laser scanning microscope

Huh7.5 cells were seeded in glass bottom dishes (35 mm dish with 14 mm bottom well) for live cell microscopy measurement. After incubation at 37 °C for 24 h, cells were treated without or with 20 μM GL22 for another 24 h. After being washed twice with PBS, cells were co-loaded with 10 μg/mL Bodipy 493/503 or 5 μM NAO and 1 μM BODIPY 558/568 C12. A laser scanning confocal microscope LSM 710 (Carl Zeiss, Oberkochen, Germany) was used for co-localization analysis.

### Analysis of mitochondrial membrane potential and apoptosis

MMP was determined using Mitochondrial Membrane Potential Detection Kit (Beyotime, China) according to the manufacturer’s instructions. Detection of GL22-induced apoptosis was performed using a commercially available Annexin V-FITC/PI apoptosis detection kit (KeyGEN, China). Briefly, Huh7.5 cells were plated into 6-well plate and treated with certain concentrations of GL22 (0, 10, 20, 30, and 40 μM). Treated cells were then harvested and stained with JC-1 or Annexin V-FITC and PI, respectively. After incubation for indicated time, cells were analyzed by flow cytometry.

### Western blotting

After incubation with GL22 (0, 20, 30, and 40 μM) for 24 h, Huh7.5 cells were collected by trypsinization and centrifugation, washed twice with ice-cold PBS and lysed with RIPA buffer. After that, protein samples were resolved on 8 or 12% SDS-PAGE gels, electro-transferred to nitrocellulose membranes and incubated with primary antibodies and secondary antibodies, and finally detected by enhanced chemiluminescence.

### Proteomic analysis

Proteomic analysis of Huh7.5 cells was conducted by PTM-Bio labs Co., Ltd. (HangZhou, China). Briefly, Huh7.5 cells seeded in 100 mm dishes were treated with 25 μM GL22 for 0, 12, and 24 h, and cells were then collected and lysed to obtain total cellular protein. Protein samples were then digested, labeled, separated, and quantified by LC-MS/MS. The bioinformatic analyses of protein annotation, functional classification, functional enrichment, and cluster analyses were then performed, and all MS data have been deposited to the ProteomeXchange Consortium via the PRIDE partner repository (data set identifier PXD006386).

### Cloning and construction of eukarya expression vectors of FABP1, FABP4, and FABP5

Total RNA was isolated from Huh7.5 cells using the TRIpure reagent (Aidlab, China) according to the manufacture’s protocol. The cDNA synthesis was carried out using a reverse transcription kit (Takara, China) with the DNase I-treated total RNA as template. The full-length cDNA sequences of FABP1, FABP4, and FABP5 were obtained by polymerase chain reaction (PCR). The PCR products were gel-purified, digested with restriction enzymes, and ligated into the pmax vector. The constructed plasmid (pmax-FABP1, pmax-FABP4, and pmax-FABP5) was transformed into *E. coli* DH5α competent cells, and the cloned gene was confirmed by sequencing.

### Transfection and identification of pmax-FABP1, pmax-FABP4, and pmax-FABP5

The expression vectors (pmax-FABP1, pmax-FABP4, and pmax-FABP5), were transfected into Huh7.5 cells using the translipofectamine (Bioino, China) according to the manufacture’s protocol. Briefly, 5 × 10^5^ of Huh7.5 cells were seeded into 6-well plates one day before transfection. Prior to transfection, all reagents were brought to room temperature (RT), and the total plasmid (3 μg) was diluted into the FBS-free RPMI-1640. Translipofectamine (9 μg/μL) was added to the diluted plasmid, and mixed immediately by vortexing. After incubation for 15 min at RT, the plasmid/translipofectamine mixture was added into the cultured cells. The transfected cells were collected at 48 h post-transfection, and proteins were extracted. Protein samples were resolved on 12% SDS-PAGE gels, electro-transferred to nitrocellulose membranes and incubated with primary antibodies against FABP1/4/5 and secondary antibodies, and finally detected by enhanced chemiluminescence.

### Statistical analysis

All data were expressed as means ± SD. Statistical analysis was performed using SPSS 17.0. Independent-sample *t*-test and single-sample *t*-test were conducted to determine the significance between groups. Differences of *P* < 0.05 were considered statistically significant.

## Electronic supplementary material

Supplementary data
