# Supplementary material for: The natural compound GL22, isolated from Ganoderma mushrooms, suppresses tumor growth by altering lipid metabolism and triggering cell death
Source: Cell Death Dis. 2018 Jun 7;9(6):689. doi: 10.1038/s41419-018-0731-6 (PMC5992186; doi:10.1038/s41419-018-0731-6)
Supplement: Supplementary file 1 — Supplementary data [file 41419_2018_731_MOESM1_ESM.docx]

**The natural compound GL22, isolated from *Ganoderma* mushrooms, suppresses tumor growth by altering lipid metabolism and triggering cell death**

**Ge Liu^1, 2, 3^, Kai Wang^3, 4^, Shan Kuang^1, 2^, Ruobing Cao^1, 2, 3^, Li Bao^3, 4^, Rui Liu^1, 2^, Hongwei Liu^3,4*^, and Chaomin Sun^1, 2*^**

^1^CAS Key Laboratory of Experimental Marine Biology, Institute of Oceanology, Chinese Academy of Sciences, Qingdao, 266071, China

^2^Laboratory for Marine Biology and Biotechnology, Qingdao National Laboratory for Marine Science and Technology, Qingdao, 266071, China

^3^University of Chinese Academy of Sciences, Beijing, 100049, China

**^4^State Key Laboratory of Mycology, Institute of Microbiology, Chinese Academy of Sciences, Beijing,** 100101, China

^*^Corresponding author

Chaomin Sun Tel.: +86 532 82898857; fax: +86 532 82898648.

E-mail address: sunchaomin@qdio.ac.cn

Hongwei Liu Tel.: +86 10 62566577; fax: +86 10 62566577.

E-mail address: liuhw@im.ac.cn

**Running title:** GL22 suppresses tumor growth


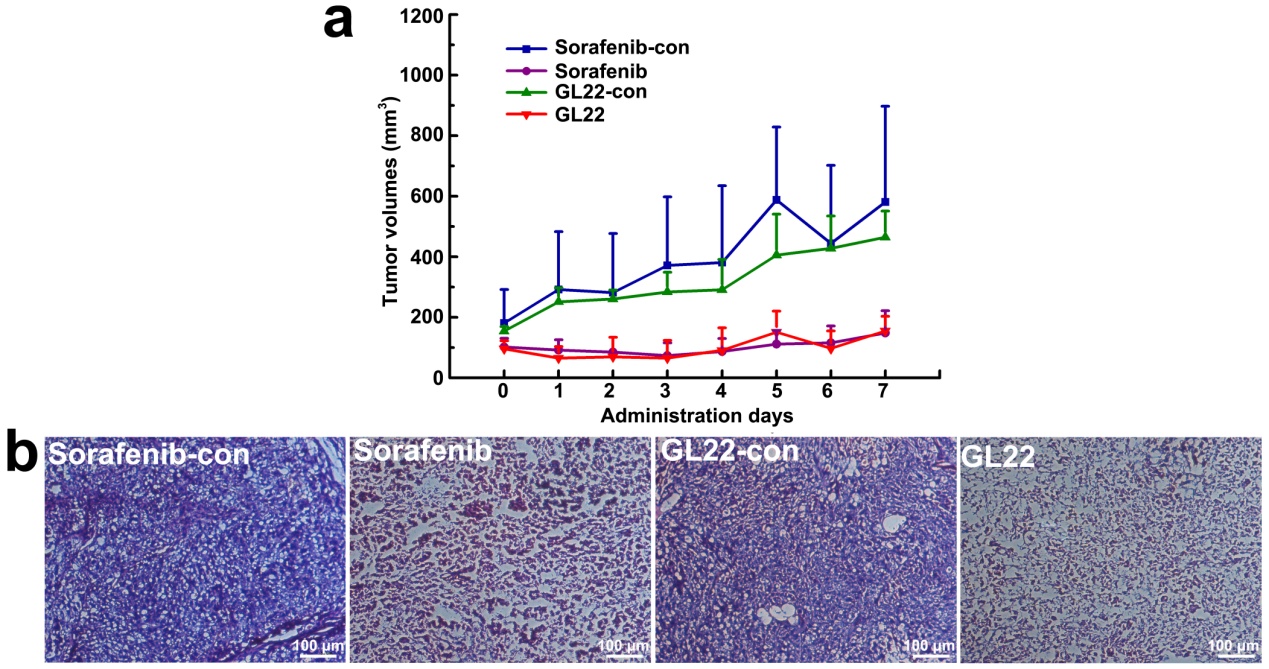


**Supplementary Fig. 1 GL22 inhibits tumor growth in Huh7.5 cell xenografts in BALB/c-nu mice**. **a** Tumor growth curves for Huh7.5 tumors of each group. n = 3 in each group. **b** Representative HE (haematoxylin and eosin) staining of the tumor tissues in xenografts in each group as following: Sorafenib-control, Sorafenib, GL22-control, GL22. Cell nuclei were counterstained with hematoxylin (blue). Scale bar = 50 μm.


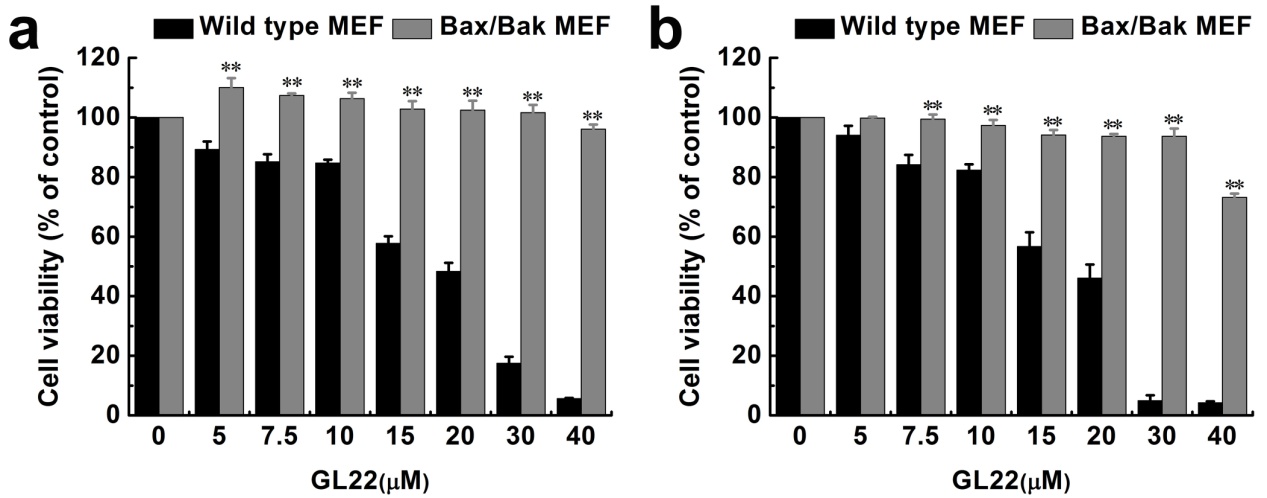


**Supplementary Fig. 2 Effects of GL22 on cell viability in wild type-MEF (mouse embryonic fibroblasts) and KO-Bax/Bak-MEF cells.** After treated with 0, 5, 7.5, 10, 15, 20, 30 and 40 μΜ GL22 for 24 h (**a**) and 48 h (**b**), cell viability was measured by MTT method.


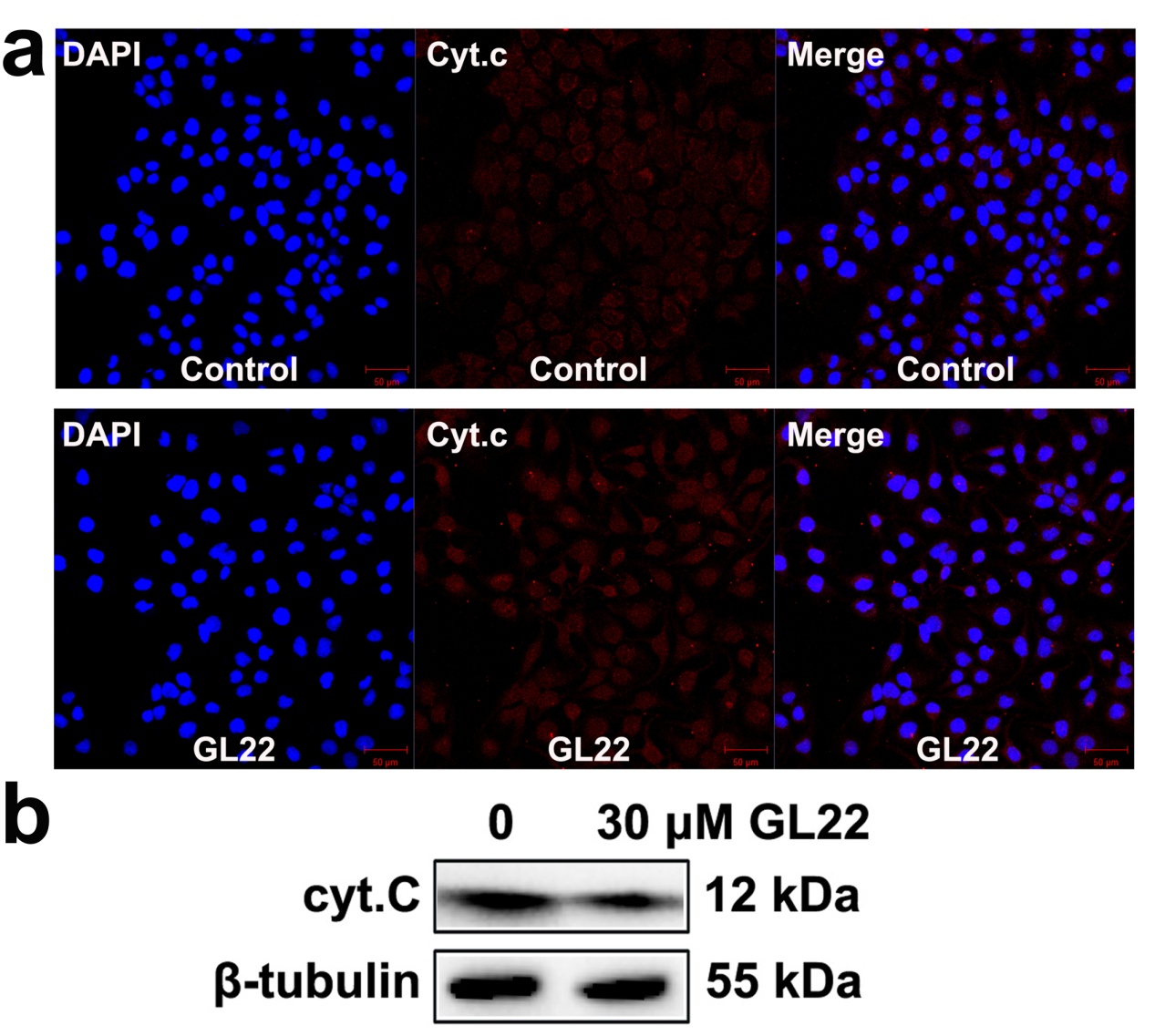


**Supplementary Fig. 3 Effects of GL22 on the release of cytochrome c in Huh7.5 cells.** After treated with 0 and 30 μΜ GL22 for 24 h, the cytochrome c in cytosol was measured by immunofluorescence staining (**a**), and the cytochrome c in mitochondria was measured by western blotting (**b**).


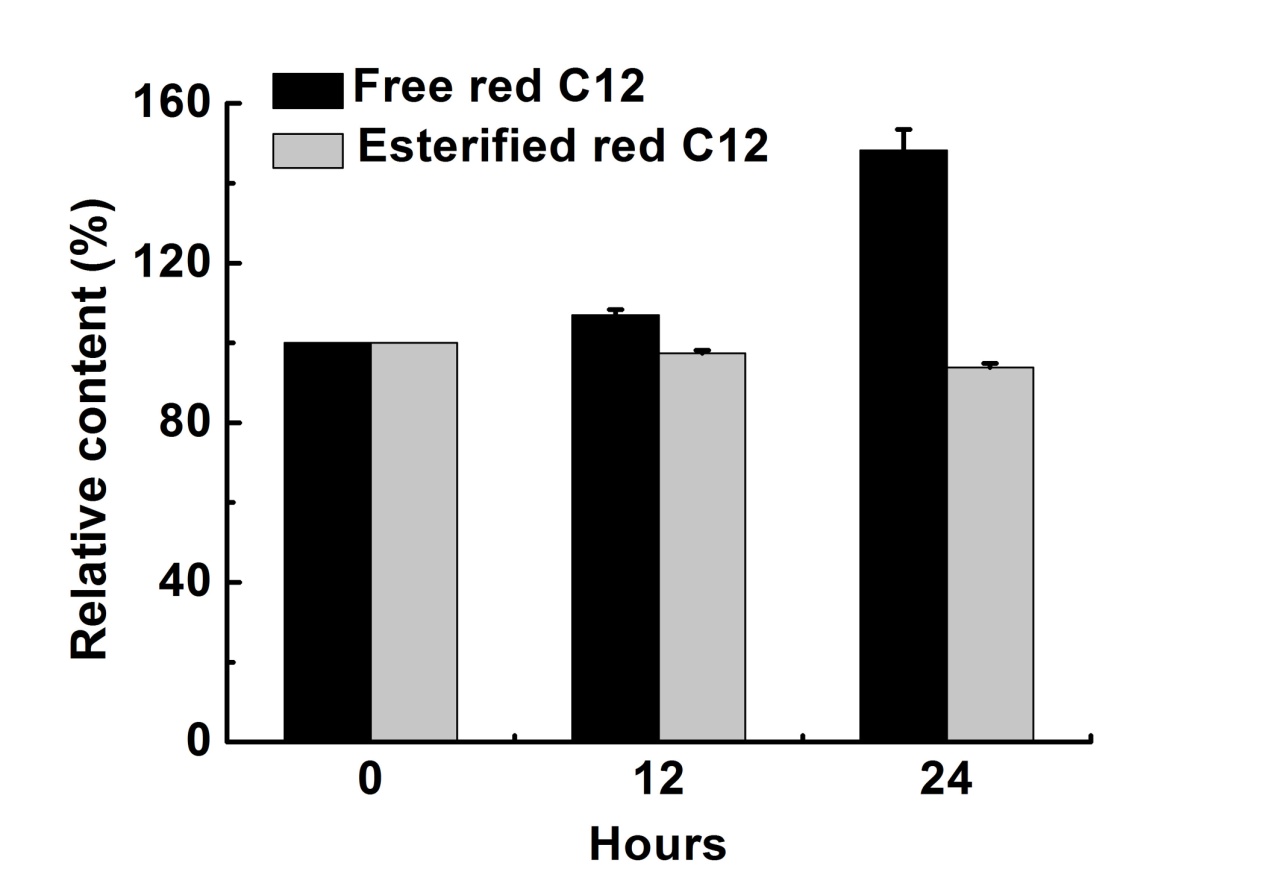
**Supplementary Fig. 4** The quantitative analysis of free /Esterified Red C12 signals. Results are normalized to untreated cells. All experiments were performed n = 3 in replicates.


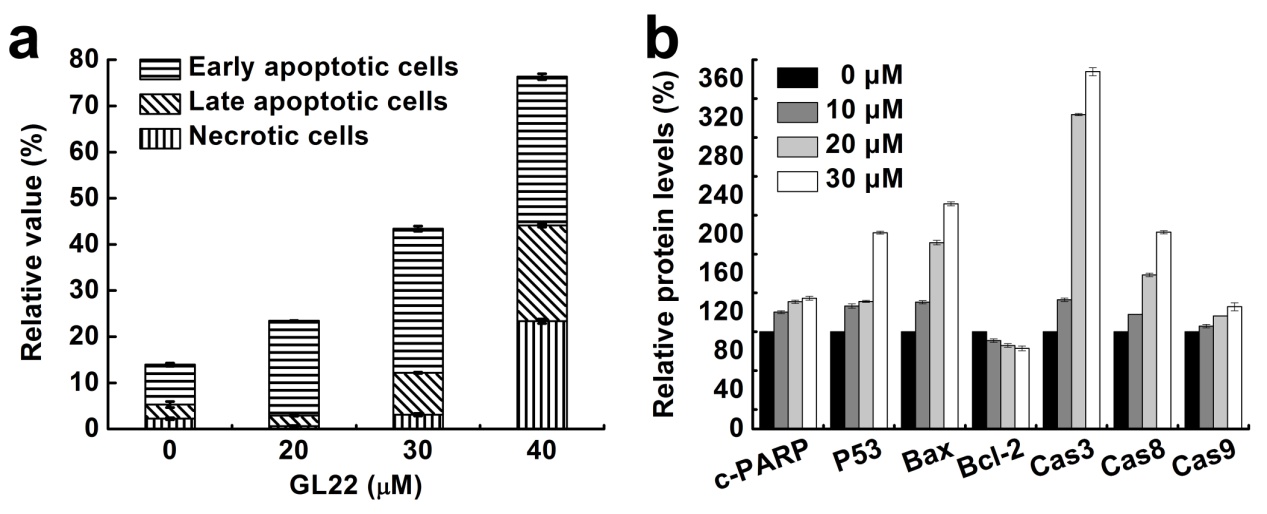


**Supplementary Fig. 5** The quantitative analyses of apoptotic cells (**a**) and apoptosis-related protein expression levels (**b**) induced by GL22 in Huh7.5 cells. Results are normalized to untreated cells. All experiments were performed n = 3 in replicates.

**Supplementary Table 1. Summary of differently expressed proteins**

| Compared sample name | Up-regulated (>1.3) | Down-regulated (<0.77) |
| --- | --- | --- |
| H12vsCON | 12 | 116 |
| H24vsCON | 19 | 122 |
| H24vsH12 | 23 | 31 |

Huh7.5 cells were treated with 25 μM GL22 for 0 (CON), 12 (H12) and 24 h (H24), and the cellular total proteins were quantified by proteomic analysis. The fold-change cutoff was set when proteins with quantitative rations above 1.3 or below 0.77 are deemed significant. *P* < 0.05.

**Supplementary Table 2. The differentially expressed proteins after 12 h treatment in Huh7.5 cells**

| Protein description | H12vsCON Ratio | *P* value |
| --- | --- | --- |
| Cytochrome P450 1A1  Zinc transporter 1  Hemoglobin subunit alpha  Coagulation factor V  Transmembrane protein 97  Transmembrane 4 L6 family member 1  15 kDa selenoprotein  GrpE protein homolog 2, mitochondrial  Non-histone chromosomal protein HMG-14  U1 small nuclear ribonucleoprotein A  Heterogeneous nuclear ribonucleoproteins C1/C2  ESF1 homolog  Gamma-glutamylcyclotransferase  Nitrilase homolog 1  Angio-associated migratory cell protein  NADH-cytochrome b5 reductase 2  Fibrinogen gamma chain  Glucose-6-phosphate isomerase  Rho GTPase-activating protein 18  EF-hand domain-containing protein D2  UDP-glucose 4-epimerase  Hematological and neurological expressed 1 protein  40S ribosomal protein S10  E3 ubiquitin-protein ligase HECTD1  Kunitz-type protease inhibitor 1  Mothers against decapentaplegic homolog 2  Phosphatidylethanolamine-binding protein 1  Rap1 GTPase-GDP dissociation stimulator 1  NEDD8-conjugating enzyme Ubc12  Keratin, type I cytoskeletal 9  Ribulose-phosphate 3-epimerase  Spermidine synthase  Interleukin-18  Alcohol dehydrogenase [NADP(+)]  Acyl-CoA-binding protein  Fatty acid-binding protein, epidermal  Branched-chain-amino-acid aminotransferase, cytosolic  Uncharacterized protein C1orf198  Dual specificity protein phosphatase 3  Cytoplasmic dynein 1 light intermediate chain 2  Endophilin-B2  ADP-ribosylation factor 3  Tubulin beta-4B chain  Nucleoside diphosphate kinase B  Destrin  Fascin  COP9 signalosome complex subunit 8  Tubulin alpha-4A chain  Secernin-1  Myotrophin  Thymidylate synthase  Vacuolar protein sorting-associated protein 16 homolog  Four and a half LIM domains protein 1  Mothers against decapentaplegic homolog 4  B-cell lymphoma/leukemia 10  Nucleoside diphosphate kinase A  Synaptojanin-2  60S ribosomal protein L7a  Beta-enolase  Ketosamine-3-kinase  Gap junction alpha-1 protein  Male-enhanced antigen 1  Pterin-4-alpha-carbinolamine dehydratase  Aryl hydrocarbon receptor  Ubiquitin-conjugating enzyme E2 N  Phosphoglyceratemutase 1  Laminin subunit alpha-5  Asparagine synthetase [glutamine-hydrolyzing]  NEDD8  Mth938 domain-containing protein  Transgelin-2  Chloride intracellular channel protein 1  L-lactate dehydrogenase A chain  Connective tissue growth factor  Formin-binding protein 1  Protein C10  Histone H1.5  Adenosine kinase  Programmed cell death protein 2  Thioredoxin  Glutamine synthetase  Guanidinoacetate N-methyltransferase  MOB kinase activator 1B  Beta-adrenergic receptor kinase 1  Tubulin beta-2B chain  Phosphomevalonate kinase  Sortilin  Agrin  Collagen alpha-1(XVIII) chain  Basement membrane-specific heparan sulfate proteoglycan core protein  Protein AMBP  Protein jagged-1  Tubulin beta-3 chain  Serotransferrin  Ubiquitin-conjugating enzyme E2 C  Cysteine protease ATG4B  Laminin subunit beta-2  Small EDRK-rich factor 2  Histone H1.0  Apolipoprotein C-II  Apolipoprotein E  N-terminal Xaa-Pro-Lys N-methyltransferase 1  Inosine triphosphate pyrophosphatase  Choline kinase alpha  Ran-specific GTPase-activating protein  Chloride intracellular channel protein 4  Nidogen-2  Collagen alpha-1(XIV) chain  Peptidyl-prolylcis-trans isomerase NIMA-interacting 4  Obscurin-like protein 1  Fibronectin  Sorting nexin-3  Death-associated protein kinase 1  Ubiquitin-like protein 5  Hydroxymethylglutaryl-CoA synthase, cytoplasmic  60S ribosomal protein L14  TRPM8 channel-associated factor 1  Dihydropyrimidinase-related protein 5  Cochlin  Dystroglycan  Fibrinogen alpha chain  Receptor-type tyrosine-protein phosphatase F  14 kDaphosphohistidine phosphatase  ADP-ribosylation factor-like protein 3  Fatty acid desaturase 2  Acyl-CoA desaturase  Mortality factor 4-like protein 1 | 2.5303  1.6307  1.61  1.5954  1.4923  1.4286  1.4205  1.4086  1.3946  1.3464  1.3258  1.3245  0.7692  0.7688  0.7682  0.767  0.7657  0.7656  0.7647  0.7635  0.7626  0.7624  0.7617  0.7617  0.7614  0.7614  0.7613  0.7613  0.7602  0.7601  0.7597  0.7592  0.7581  0.757  0.7566  0.7563  0.7557  0.7557  0.755  0.7546  0.7544  0.754  0.7521  0.75  0.7491  0.7483  0.7483  0.7464  0.7463  0.7458  0.7457  0.7451  0.745  0.7443  0.7435  0.7432  0.7428  0.7424  0.7415  0.7413  0.7404  0.7383  0.7376  0.7373  0.7343  0.7331  0.7326  0.7317  0.7309  0.7306  0.7292  0.7288  0.7276  0.7261  0.7256  0.7232  0.7228  0.7189  0.7169  0.7145  0.7138  0.7132  0.7131  0.7123  0.7119  0.7093  0.7078  0.7055  0.7027  0.6968  0.6957  0.6942  0.6941  0.6935  0.6932  0.6918  0.69  0.6884  0.6883  0.6876  0.6868  0.6859  0.6858  0.6829  0.6775  0.6745  0.6691  0.6686  0.6677  0.6633  0.6614  0.6539  0.6529  0.6482  0.6457  0.6447  0.6393  0.6391  0.6358  0.6293  0.6227  0.6224  0.6208  0.6168  0.5903  0.5666  0.548 | 0.000515027  0.019904439  9.19185E-05  7.39403E-07  1.06786E-06  0.002199523  0.025776898  0.000308877  0.004386407  2.70658E-06  1.92113E-12  5.03676E-06  1.79533E-06  0.000736366  0.000222132  0.00458249  0.000855044  9.91012E-07  2.69562E-12  0.000300748  2.7518E-07  0.025615492  4.99854E-09  1.09068E-12  0.000173109  0.000925013  0.014636218  0.018542962  4.38599E-07  0.00746388  0.000207322  3.69578E-07  3.21167E-05  8.64138E-06  0.000759799  9.18018E-08  0.032063447  1.42273E-05  0.001981649  0.00960311  0.000641849  3.44014E-08  0.001436239  4.28191E-08  0.003985284  4.83765E-10  0.000638774  0.000135032  0.000695052  0.007592866  0.001962032  0.040018491  0.002820541  0.004614552  0.003686908  1.81196E-07  0.008465286  4.75168E-07  1.38556E-13  0.001257541  0.029515072  0.032614576  0.021578936  0.016049394  2.99585E-07  8.65752E-13  3.25429E-12  3.8359E-08  0.007328384  0.008027299  1.01012E-08  6.90609E-10  0  7.90151E-05  0.011883078  0.000978887  0.000332821  0.030800162  0.004822284  8.02053E-08  0.006879668  0.002160661  0.003098842  2.93309E-05  0.005385154  0.000139103  0.036328329  2.45452E-07  0.000460017  0  0.000133116  2.31561E-11  0.007652851  3.15303E-14  0.003416648  0.003590511  0.037198181  0.013251158  0.000506855  0.016416176  3.90656E-05  0.04277074  0.02746012  0.002026332  2.37851E-06  2.23401E-05  3.69241E-06  3.14488E-11  0.013306705  0.00089523  1.3034E-13  0.000507593  0.003908832  0.013430648  1.11466E-13  0.000459607  4.38872E-08  0.003056611  0.002577605  0.006477745  7.71952E-06  7.58727E-09  0.016902909  0.034537687  4.90192E-08  9.63622E-08  0.002908202 |
| Neuronal cell adhesion molecule | 0.4167 | 1.09343E-07 |

**Supplementary Table 3. The differentially expressed proteins after 24 h treatment in Huh7.5 cells**

| Protein description | H24vsCON Ratio | *P* value |
| --- | --- | --- |
| Cytochrome P450 1A1  Hemoglobin subunit alpha  Coagulation factor V  Zinc transporter 1  Transmembrane 4 L6 family member 1  Interferon-related developmental regulator 1  Coiled-coil domain-containing protein 86  Zinc finger protein OZF  Serine/arginine-rich splicing factor 6  Surfeit locus protein 6  Enhancer of polycomb homolog 1  Insulin receptor substrate 2  Mitochondrial fission 1 protein  Nucleoplasmin-3  Serine/arginine-rich splicing factor 4  U1 small nuclear ribonucleoprotein A  Complement decay-accelerating factor  Cell growth-regulating nucleolar protein  Four and a half LIM domains protein 2  Signal transducing adapter molecule 2  Sorting nexin-3  Fatty acid synthase  Lanosterol 14-alpha demethylase  EF-hand domain-containing protein D2  Ran-specific GTPase-activating protein  Protein EFR3 homolog A  Alpha-taxilin  NADH dehydrogenase [ubiquinone] iron-sulfur protein 8, mitochondrial  RNA-binding protein with multiple splicing  Inhibin beta E chain  Proliferating cell nuclear antigen  Thioredoxin domain-containing protein 9  Myotrophin  Angio-associated migratory cell protein  Coiled-coil-helix-coiled-coil-helix domain-containing protein 2  Deoxyhypusine synthase  Beta-adrenergic receptor kinase 1  Catenin delta-2  Sterol 26-hydroxylase, mitochondrial  Acyl-CoA-binding protein  Collagen alpha-2(V) chain  Coiled-coil domain-containing protein 80  ADP-ribosylation factor 3  Clustered mitochondria protein homolog  Single-stranded DNA-binding protein, mitochondrial  Kinesin-like protein KIFC3  E3 ubiquitin-protein ligase HECTD1  Heat shock factor-binding protein 1  Alpha-fetoprotein  Caprin-1  Prolyl 3-hydroxylase 2  Cytoplasmic dynein 1 light intermediate chain 2  Monocyte differentiation antigen CD14  NADH dehydrogenase [ubiquinone] iron-sulfur protein 7, mitochondrial  Cystathionine beta-synthase  Phosphatidylserine decarboxylase proenzyme  Low-density lipoprotein receptor-related protein 2  Protein BRICK1  Muskelin  Protein C10  Angiomotin-like protein 2  Protein jagged-1  Peptidyl-prolylcis-trans isomerase NIMA-interacting 4  45 kDa calcium-binding protein  Ubiquitin-conjugating enzyme E2 C  Endothelial differentiation-related factor 1  Synaptojanin-2  Eukaryotic translation initiation factor 1  MOB kinase activator 1B  Cytochrome c oxidase copper chaperone  Glutamine synthetase  NADH dehydrogenase [ubiquinone] 1 alpha subcomplex subunit 7  39S ribosomal protein L2, mitochondrial  Laminin subunit beta-1  Calponin-3  Collectin-12  Synaptotagmin-like protein 5  Endonuclease G, mitochondrial  Chloride intracellular channel protein 4  Fibroblast growth factor receptor 4  14 kDaphosphohistidine phosphatase  Formin-binding protein 1  CCAAT/enhancer-binding protein alpha  NADH dehydrogenase [ubiquinone] 1 alpha subcomplex subunit 2  Cysteine-rich motor neuron 1 protein  Lymphoid-specific helicase  Thymidylate synthase  NADH dehydrogenase [ubiquinone] iron-sulfur protein 6, mitochondrial  AN1-type zinc finger protein 6  Mothers against decapentaplegic homolog 4  Male-enhanced antigen 1  Plasminogen activator inhibitor 1  Laminin subunit gamma-1  Cytochrome c oxidase subunit 7A-related protein, mitochondrial  Apolipoprotein E  Endothelin-converting enzyme 1  NADH dehydrogenase [ubiquinone] flavoprotein 1, mitochondrial  Transcription factor SOX-13  Asialoglycoprotein receptor 1  NADH-ubiquinone oxidoreductase 75 kDa subunit, mitochondrial  Store-operated calcium entry-associated regulatory factor  Glypican-3  Clusterin  Collagen alpha-1(XVIII) chain  Fibrinogen gamma chain  Amyloid beta A4 protein  Kunitz-type protease inhibitor 1  Choline kinase alpha  Collagen alpha-1(XVI) chain  Obscurin-like protein 1  Dystroglycan  Apolipoprotein C-I  Gap junction alpha-1 protein  Ubiquitin-like protein 5  Nidogen-1  Fibrinogen-like protein 1  Connective tissue growth factor  Small EDRK-rich factor 2  Fibronectin  Laminin subunit alpha-5  Receptor-type tyrosine-protein phosphatase F  Protein AMBP  Sortilin  Hydroxymethylglutaryl-CoA synthase, cytoplasmic  Basement membrane-specific heparan sulfate proteoglycan core protein  Serotransferrin  Death-associated protein kinase 1  Apolipoprotein C-II  Mortality factor 4-like protein 1  TRPM8 channel-associated factor 1  Thymosin beta-10  Laminin subunit beta-2  Agrin  Collagen alpha-1(XIV) chain  Nidogen-2  Cochlin  Angiopoietin-related protein 3  Fibrinogen alpha chain  Neuronal cell adhesion molecule  Acyl-CoA desaturase | 2.1402  1.9302  1.6858  1.6825  1.5059  1.4689  1.4256  1.386  1.3608  1.36  1.3584  1.3318  1.3294  1.3286  1.3275  1.3268  1.3225  1.3136  1.3046  0.7692  0.7669  0.7663  0.7663  0.766  0.7658  0.7657  0.7651  0.764  0.7638  0.7637  0.7635  0.7626  0.7619  0.7614  0.7609  0.7602  0.7601  0.7596  0.7594  0.7583  0.758  0.7576  0.7573  0.7571  0.7549  0.7545  0.7533  0.7511  0.75  0.75  0.7498  0.7463  0.7458  0.7449  0.7435  0.7434  0.7422  0.7392  0.738  0.7379  0.7373  0.7371  0.734  0.7335  0.7275  0.7275  0.7272  0.7239  0.7237  0.7226  0.7211  0.7159  0.7147  0.7121  0.712  0.7112  0.7094  0.7062  0.7053  0.7018  0.7012  0.6998  0.6993  0.6959  0.6935  0.6914  0.6911  0.6906  0.6904  0.6877  0.6874  0.6799  0.6749  0.6736  0.6701  0.6686  0.6664  0.6644  0.6613  0.6603  0.6602  0.6598  0.6596  0.659  0.653  0.6503  0.6474  0.6473  0.6459  0.6419  0.6392  0.6358  0.6075  0.6069  0.6007  0.5977  0.5968  0.5825  0.571  0.5649  0.5545  0.553  0.5493  0.5401  0.536  0.5347  0.5301  0.5289  0.5199  0.5152  0.5143  0.4967  0.4952  0.4928  0.4895  0.4723  0.466  0.4566  0.4185  0.3452 | 0.001036401  9.40985E-06  1.08017E-06  0.022874623  0.004081257  0.000717742  3.93696E-08  0.001721107  1.98266E-08  1.48009E-09  0.023471203  0.000120476  0.01257134  0.012303134  0.000159379  4.03571E-05  0.000174581  8.06022E-13  6.33648E-08  1.11952E-05  0.004207931  0  7.11322E-11  2.83677E-05  2.23976E-05  0.005496094  1.21477E-05  0.045209385  0.0155372  0.001738821  8.60881E-07  0.013117328  0.01568641  0.000423403  0.012926896  0.024720915  2.87219E-05  1.81894E-06  0.011693482  0.000276045  0.011754524  0.039169891  7.68292E-09  0.000133408  5.14115E-10  0.022519332  4.15734E-12  0.000532375  0.000338907  7.7234E-10  5.63799E-06  0.012638859  0.034181527  0.000144493  0.000539331  0.005934527  0.003347539  0.008052135  0.023066835  0.000268999  0.001187982  1.53746E-09  0.01979893  1.36845E-06  0.003986593  4.79687E-07  0.005648739  0.011426864  0.004143548  0.003076965  0.001155965  0.015038607  0.005281995  6.7506E-12  8.5838E-12  0.009621283  0.028085132  0.001092779  2.89793E-05  0.033575456  0.027598921  0.004877938  0.03146532  2.84344E-05  3.86329E-05  2.40036E-09  0.002438615  0.009941015  0.024399276  0.001471153  0.017114782  0.040534001  0  0.008755322  5.01931E-06  0.042865038  2.8873E-09  2.2734E-07  0.001242034  7.27795E-10  0.02671076  7.45225E-06  5.55357E-07  3.84536E-05  0.000798965  0.014835025  5.29216E-06  0.001162082  0.005658064  0.000515892  0.003078971  0.016119876  0.021908374  0.008985685  2.95613E-07  0.001107449  3.69748E-07  0.017546762  0  0  1.53973E-09  1.76531E-05  0.006570438  0  0  0  0.002364747  0.002182265  0.000880828  1.71037E-11  0.041463059  0.023600439  7.6894E-12  0  3.02966E-09  0.00332553  0.010686241  7.50542E-07  4.11558E-08  2.35049E-09 |
| Fatty acid desaturase 2 | 0.3238 | 2.9617E-09 |


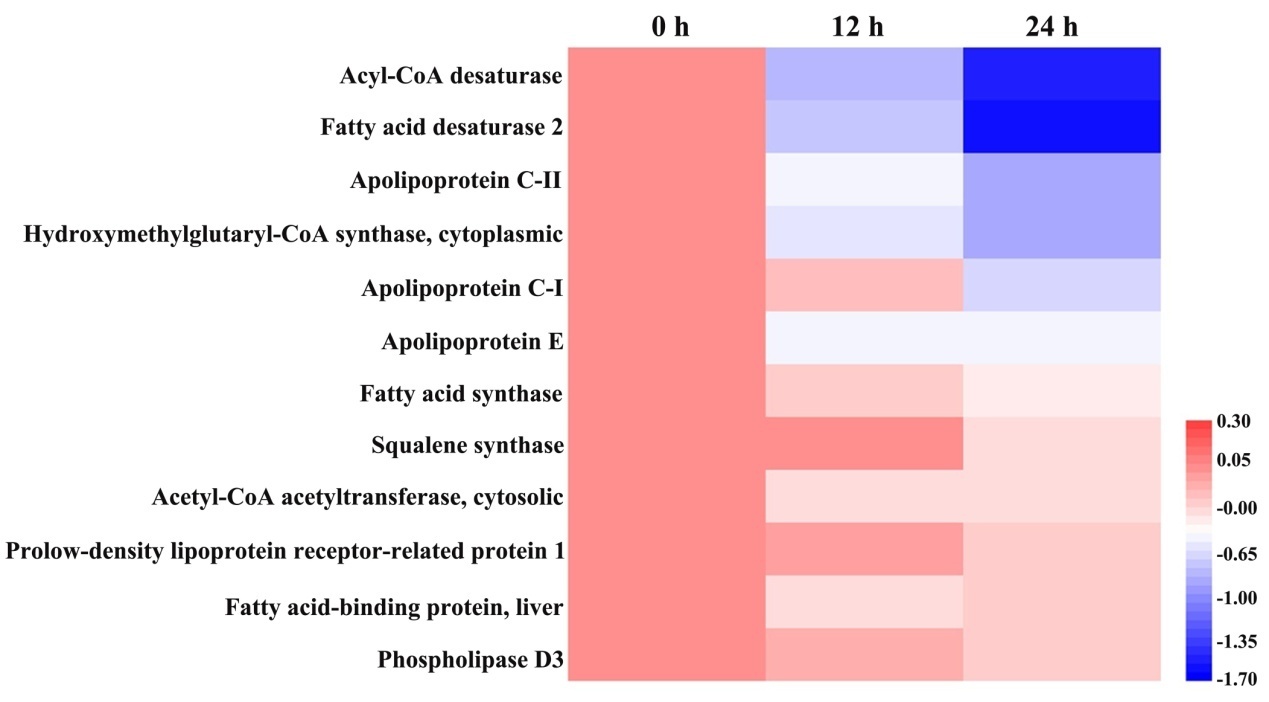
**Supplementary Fig. 6 Effect of GL22 on the expressions of proteins associated with fatty acid in Huh7.5 cells**. Huh7.5 cells were treated with 25 μM GL22 for 0, 12 and 24 h and then proteins were extracted, separated and identified using LC-ESI-MS/MS analyses. The relative protein abundances of differentially expressed proteins related to fatty acid (fold change ≥1.3 or fold change ≤0.77 and *P* value <0.05) were imported for clustering analysis using HemI.


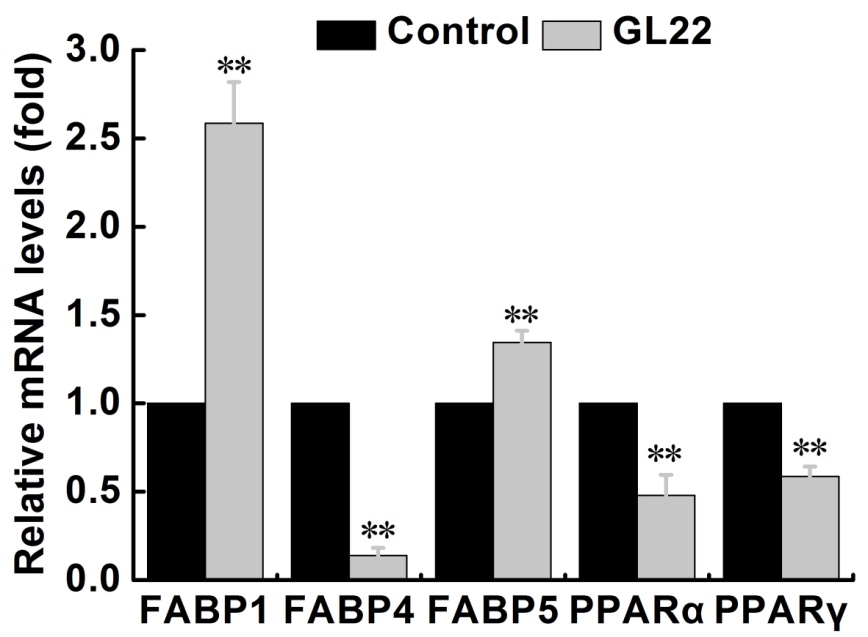


**Supplementary Fig. 7** The effect of GL22 on the transcriptional levels of FABP1, FABP4, FABP5, PPARα, PPARγ, after Huh7.5 cells were treated with 0 and 20 μΜ GL22 for 24 h.


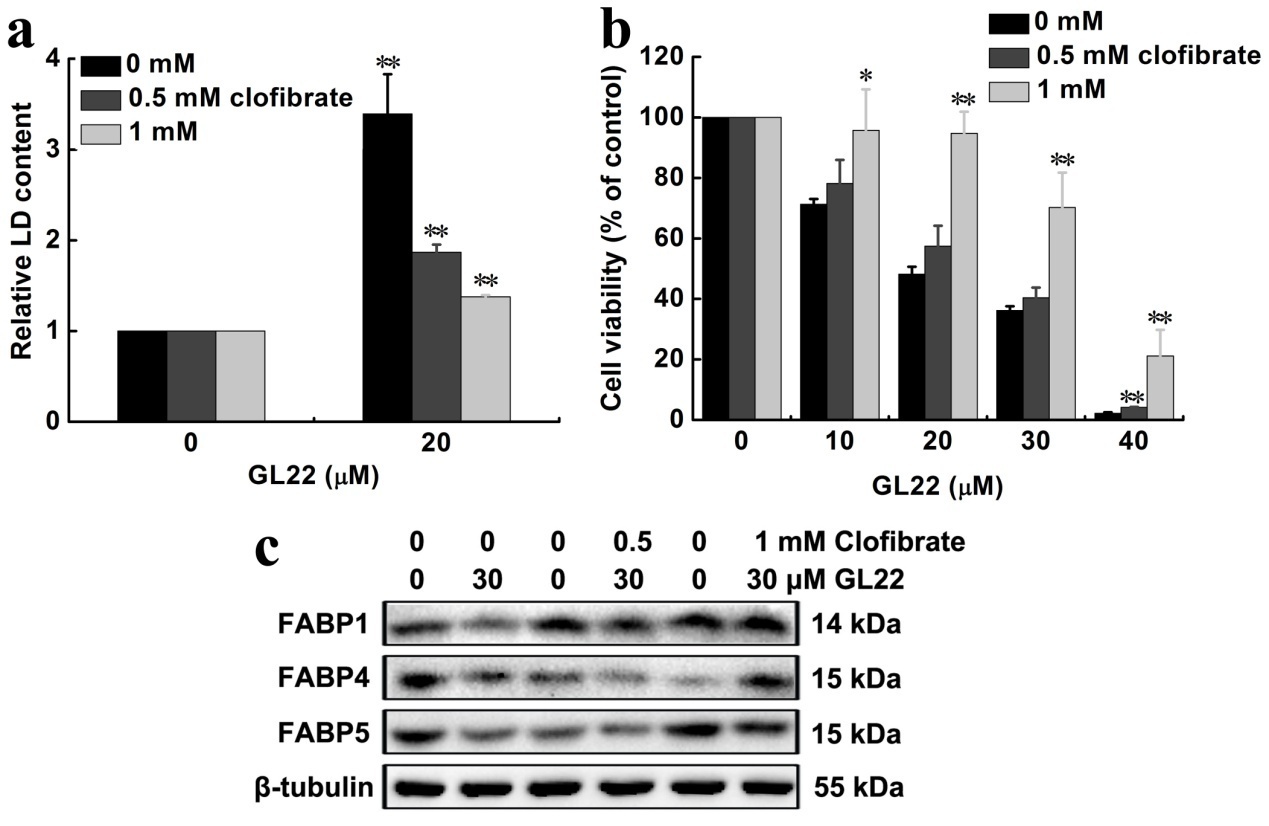


**Supplementary Fig. 8 Clofibrate (a PPAR agonist) rescued the accumulation of LDs, decrease of cell viability and reductions of FABPs expression levels induced by GL22. a** Huh7.5 cells were pretreated with 0.5 or 1 mM clofibrate for 1 h, and then 20 μΜ GL22 was added for another 12 h. LD production was measured by flow cytometry. **b** Huh7.5 cells were pretreated with 0.5 or 1 mM clofibrate for 1 h, and then GL22 (0, 10, 20, 30 and 40 μΜ) was added for another 48 h. Cell viability was measured by MTT method. **c** 0.5 or 1 mM clofibrate was pre-incubated with cells for 1 h, and then treated with 0 and 30 μΜ GL22 for another 24 h.


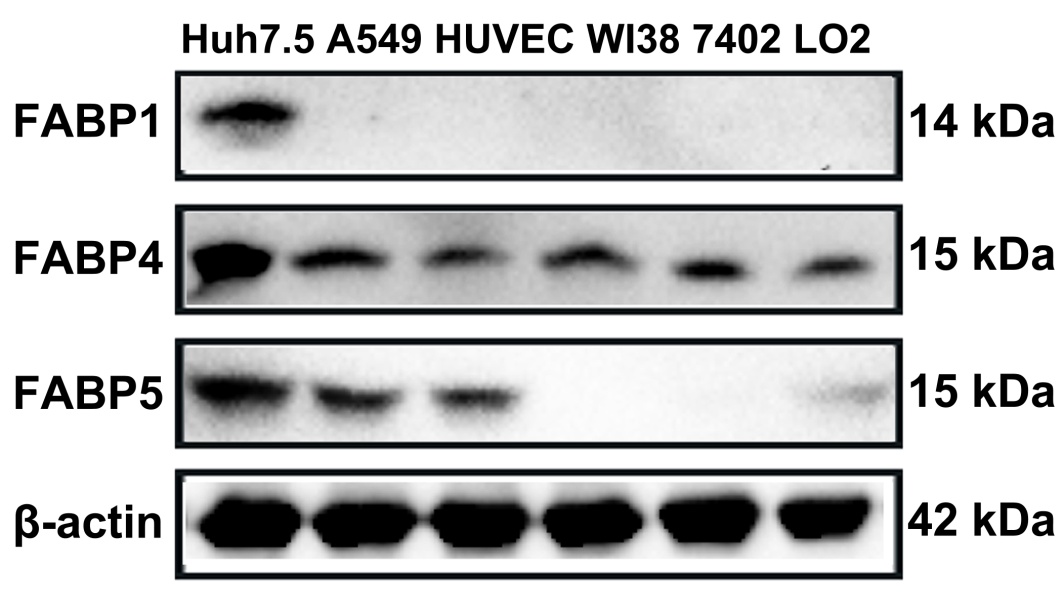


**Supplementary Fig. 9 The difference in expression profiles of FABP1, FABP4 and FABP5 among six human cell lines.** Human liver cancer cell line (Huh7.5), human lung cancer cell line (A549), human umbilical vein endothelial cell (HUVEC),normal human fetal lung fibroblast (WI38), human liver cancer cell line (Bel-7402), and normal human liver cells (LO2) were harvested by trypsinization and centrifugation, washed twice with ice-cold PBS, and then proteins were extracted using RIPA buffer. After that, protein samples were resolved on 12% SDS-PAGE gels, electro-transferred to nitrocellulose membranes and incubated with primary antibodies and secondary antibodies, and finally detected by enhanced chemiluminescence.

| **Supplementary Table 4. Nucleotide sequences of the primers used in the experiments** | | | |
| --- | --- | --- | --- |
|  | Primers |  | Base pairs |
| P1 | FABP1 primer  forward | 5’-cggggtaccatgagtttctccggcaag -3’ | 27 |
| P2 | FABP1 primer  reverse | 5’-ccgctcgagttaaattctcttgctgattc -3’ | 29 |
| P3 | FABP4 primer  forward | 5’-ctagctagcatgtgtgatgcttttgtag -3’ | 28 |
| P4 | FABP4 primer  reverse | 5’-ccgctcgagttatgctctctcataaac -3’ | 27 |
| P5 | FABP5 primer  forward | 5’- cggggtaccatggccacagttcagc -3’ | 25 |
| P6 | FABP5 primer  reverse | 5’- ccgctcgagttattctactttttcatag -3’ | 28 |

Total RNA was isolated from Huh7.5 cells using TRIpure reagent (Aidlab, China) according to the manufacture’s protocol. The cDNA synthesis was carried out using a reverse transcription kit (Takara, China) with the DNase I-treated total RNA as template. The full-length cDNA sequences of FABP1, FABP4 and FABP5 were obtained by polymerase chain reaction (PCR) using the oligonucleotide primers in the table.
